# An observational study on lifestyle and environmental risk factors in patients with acute appendicitis

**DOI:** 10.1016/j.heliyon.2023.e15131

**Published:** 2023-04-01

**Authors:** Toon Peeters, Bert Houben, Peter Cools, Yati Thys, Valentino D'Onofrio, Sandrina Martens, Martin Jaeger, Marije Doppenberg-Oosting, Mihai G. Netea, Inge C. Gyssens

**Affiliations:** a Department of Infectious Diseases and Immunity, Jessa Hospital, 3500 Hasselt, Belgium; b Department of Experimental Pathology, Vrije Universiteit Brussel (VUB), 1090 Brussels, Belgium; c Department of Internal Medicine, Radboud University Medical Center, 6525 GA Nijmegen, the Netherlands; d Radboudumc Center for Infectious Diseases (RCI), Radboud University Medical Center, Nijmegen, the Netherlands; e Department of Abdominal and Oncological Surgery, Jessa Hospital, 3500 Hasselt, Belgium; f Department of Abdominal Surgery, GZA Hospital, Antwerp, Belgium; g Radboud Institute for Molecular Life Sciences, Radboud University Medical Center, Nijmegen, the Netherlands

**Keywords:** Appendicitis, Environment, Demography

## Abstract

**Purpose:**

Acute appendicitis is a common abdominal emergency worldwide. This study aimed at characterizing environmental risk factors influencing the development and severity of acute appendicitis.

**Methods:**

Patients from a Belgian acute appendicitis cohort (n = 374) and healthy controls from the 500 functional genomics (500FG) cohort (n = 513) were compared. Individuals with a history of appendectomy (n = 1067) and without a history of appendectomy (n = 8656) were available from the Nijmegen Biomedical Study (NBS). Questionnaires on demographics, lifestyle and environment were available. Binary logistic regression was used for prediction models.

**Results:**

Fifteen risk factors for developing acute appendicitis were identified. Binary logistic regression showed that 7 were independent risk factors: family history of acute appendicitis, having grown up in a rural environment, having a lower education, probiotic use as well as antibiotic use increased the risk of developing appendicitis. Fruit and fiber-rich vegetable consumption decreased the risk. Findings on vegetable consumption, smoking and level of education were replicated in the NBS population. Independent risk factors for complicated appendicitis were being male, higher age, and a delay to diagnosis of more than 48 h.

**Conclusions:**

Environmental exposures influence the risk of developing appendicitis. Further research into these factors is needed.

## 1 Introduction

Acute appendicitis is one of the most common surgical emergencies worldwide [1]. The lifetime risk is estimated between 6 and 17%, mostly depending on geographic region [2,3]. The disease can be associated with serious complications, such as perforation, abscess formation and peritonitis. Based on these complications, appendicitis is classified as complicated and uncomplicated. Based on the presence of necrosis in the appendix tissue, the disease can also be classified as gangrenous or non-gangrenous.

The etiology of acute appendicitis is likely multifactorial. Certain environmental exposures have been associated with appendicitis, which are often related to a Western-type lifestyle. The effects of diet on the risk of developing acute appendicitis have been studied extensively in the past [4,5]. Hygiene has also been associated with appendicitis, as improvements in hygiene appear to be connected to a higher incidence of the disease [6,7]. Appendicitis is also more common in smokers [8], and incidence appears to be higher during the summer, which according to the authors may partly be explained by higher temperatures and more air pollution during this period [9]. However, gastrointestinal infections in general have higher incidences during the summer [10].

Incidence of appendicitis varies among regions in the world, and is lowest in low-income countries, and higher in recently industrialized countries [11]. The incidence also fluctuated over time, with an increase in Western Countries at the start of 20th century, reaching a peak mid-century, followed by a decrease towards the end [11]. Since then, incidence has remained stable.

This study aimed at identifying environmental and lifestyle-related factors contributing to the risk of developing acute appendicitis.

## 2 Patients and methods

### 2.1 Study population

**Fig. 1:**
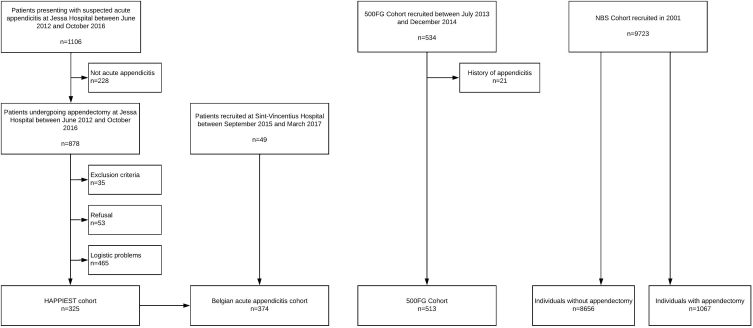
Flowchart of the patient and control recruitment in the Hasselt APPendicitis Immunologic and Environmental STudy (HAPPIEST), the 500 Functional Genomics (500FG) population and the Nijmegen Biomedical Study (NBS).

Fig. 1 shows a flowchart of the different study populations. A total of 325 patients were prospectively recruited at Jessa Hospital, Hasselt, Belgium in the Hasselt APPendicitis Immunologic and Environmental STudy (HAPPIEST). An additional 49 patients were recruited at Sint-Vincentius hospital, Antwerp, Belgium. Patients from both populations were taken together into the Belgian acute appendicitis cohort. Patients received standard care. Following removal, the appendix was sectioned by the surgeon and classified according to the International Classification of Diseases (ICD)-9 codes. Appendicitis with generalized peritonitis (540.0) or peritoneal abscess (540.1) was considered complicated, appendicitis with no mention of peritonitis or abscess (540.9) was considered uncomplicated. After sectioning of the appendix, a 1 cm section of the tip, the middle and the base were sent to the pathology department to assign the histological severity of appendicitis as gangrenous vs non-gangrenous, where gangrenous appendicitis is defined by the presence of severe transmural inflammation and areas of necrosis. Exclusion criteria for acute appendicitis were appendectomy more than 5 days after onset of symptoms. Patients with acute appendicitis between the ages of 5 and 85 were considered eligible. Pregnant and immunocompromised patients were also excluded in order to obtain a homogenous population. Clinical data and medical history of patients who underwent surgery for acute appendicitis were recorded. Five hundred and thirteen healthy control subjects were selected from the 500 Functional Genomics (500FG) cohort (n = 534), which mainly consisted of university students. Selected controls had no history of acute appendicitis. The 500FG population was recruited at Radboud university medical center, Nijmegen, the Netherlands [12]. Patients and controls were recruited between 2012 and 2017.

Another series of 1067 individuals with a self-reported history of appendectomy and 8656 controls with no history of appendectomy was obtained from the Nijmegen Biomedical Study (NBS) in 2001. Details of this study have been reported before [13]. This cohort was used in order to validate findings in the Belgian acute appendicitis cohort versus the 500FG controls. The NBS was a population-based study, and history of appendectomy spanned over a period of approximately 40 years. Since over a time period of 40 years lifestyle and environment may change, a selection of NBS individuals with a history of appendectomy in the last 15 years before questioning was made for comparison with the Belgian acute appendicitis population.

Patients and controls filled out questionnaires on potential determinants. Questionnaires covered demographic data, lifestyle characteristics, such as diet and smoking habits, and environmental exposures.

The lifestyle and diet questionnaires used in the acute appendicitis and 500FG populations were very similar, and based on two previous publications from the European Prospective Investigation into Cancer and Nutrition [14,15]. Questions on smoking habits were removed from questionnaires for patients under the age of 16. The variable alcohol consumption could not be used in the comparison between these populations due to different wording of the questions.

The questionnaires for the NBS population contained questions on, among others, demographics, lifestyle, medical history, general health, use of medication and quality of life. However, since the purpose of this population for the current study was to verify results from the analyses on the Belgian acute appendicitis and 500FG populations, only data on demographics and lifestyle were analyzed.

For a comparison between the Belgian acute appendicitis patients and 500FG controls with the NBS population, answers to some of the multiple choice questions were grouped in order to match data from different questionnaires. Supplementary table S1 shows a description of both questionnaires concerning the data used for this analysis.

### 2.2 Statistical analysis

Characteristics and potential environmental determinants of patients versus controls as well as complicated versus uncomplicated, and gangrenous versus non-gangrenous appendicitis patients were compared using T-tests in case of continuous variables and χ^2^ tests in case of categorical variables. A p-value <0.050 was considered statistically significant. Considering the possible impact of age and gender on lifestyle related factors, patients from the Belgian population and controls from the 500FG population, and individuals with and without a history of appendectomy from the NBS population, were matched for gender and age, with a tolerance of 5 years. For binary logistic regression, a cut-off value of p < 0.050 was used in selection of variables. For analyses on the Belgian acute appendicitis and 500FG cohorts, an additional category of “never” was included for meat consumption and duration of breastfeeding in order to include all eligible individuals. For a number of variables, categories were grouped to prevent low numbers for certain levels.

## 3 Results

### 3.1 Characteristics of patients (Belgian acute appendicitis cohort) and controls (500FG population)

Significantly more patients were male (52.9%) and the average age of patients was higher (32.8 versus 28.3 years). Out of 114 complicated and 116 gangrenous cases, only 57 were both complicated and gangrenous.

Patient and control characteristics after matching for age and gender are summarized in Table 1. Patients more often lived with family or communities and in rural areas, whereas controls were more often single and lived in urban areas, which is also most likely a consequence of student housing. More control individuals were still studying, which is also likely a consequence of recruitment methods. Strikingly, more patients had a family history of acute appendicitis (55.8 and 6.7% respectively). These numbers are however likely influenced by information bias. Patients more often spent their youth in rural areas. The large number of control subjects not living with pets is also most likely a consequence of student housing. Patients were breastfed significantly less than controls (69.1 and 85.3% respectively), and those who were, were breastfed for a shorter period of time. Patients consumed significantly less fruit as well as less fiber-rich vegetables. Patients also consumed more sugar containing drinks, antibiotics and probiotics, and were more often smokers.

**Table 1:** Characteristics of patients (acute appendicitis cohort) and controls (500FG population) matched 1:1 for age and gender.

Demographics	Patients (n = 254)	Controls (n = 254)	p-value
	n (%)	n (%)	
**Gender**			1.000
Female	123 (48.4)	123 (48.4)	
Male	131 (51.6)	131 (51.6)	
Age, Mean ± SD (Range)	33.14 ± 15.75 (13–74)	33.22 ± 93 (18–73)	0.958
**Ethnicity**			0.564
European	236 (96.7)	248 (97.6)	
North African	4 (1.6)	1 (0.4)	
Sub-Saharan African	1 (0.4)	1 (0.4)	
Asian	3 (1.2)	4 (1.6)	
Missing	10	0	
**Marital Status**			<0.001
Single	27 (11.3)	59 (23.2)	
Living with partner, family or community	213 (88.8)	195 (76.8)	
Missing	14	0	
**Living Area**			<0.001
Rural	92 (37.9)	29 (11.4)	
(Sub)urban	151 (62.1)	225 (88.6)	
Missing	11	0	
**Education**			<0.001
Primary school	8 (3.3)	9 (3.5)	
Secondary school	76 (31.4)	36 (14.2)	
Higher education	92 (38.0)	115 (45.3)	
Studying	66 (27.3)	94 (37.0)	
Missing	12	0	
**Acute appendicitis**			
**Family history of acute appendicitis**			<0.001
Yes	129 (55.8)	13 (6.7)	
No	102 (44.2)	182 (93.3)	
Missing	23	59	
**Exposures**			
**Living area in youth**			0.011
Rural	115 (47.5)	92 (36.2)	
Urban	127 (52.5)	162 (63.8)	
Missing	12	0	
**Contact with farm animals during youth**			0.670
Seldom or never	149 (61.3)	151 (59.4)	
Daily to monthly	94 (38.7)	103 (40.6)	
Missing	11	0	
**Living with pets**			<0.001
Yes	136 (55.3)	79 (31.1)	
No	110 (44.7)	175 (68.9)	
Missing	8	0	
**Breastfeeding**			<0.001
No	63 (30.9)	33 (14.7)	
Yes	141 (69.1)	192 (85.3)	
Don't know	37	28	
Missing	13	1	
**Duration**			0.009
0–3 months	34 (39.1)	21 (19.3)	
3–6 months	29 (33.3)	50 (45.9)	
more than 6 months	24 (27.6)	38 (34.9)	
Don't know	51	59	
Missing	3	25	
**Vegan/vegetarian**			0.878
Yes	9 (3.7)	10 (3.9)	
No	236 (96.3)	244 (96.1)	
Missing	19	0	
**Meat consumption**			0.027
Daily	179 (76.8)	146 (65.5)	
Weekly	49 (21.0)	71 (31.8)	
Monthly	5 (2.1)	6 (2.7)	
Don't know	0	1	
Missing	3	0	
**Fruit consumption**			<0.001
Daily	96 (39.3)	153 (60.2)	
Weekly	107 (43.9)	88 (34.6)	
Monthly	25 (10.2)	10 (3.9)	
Never	16 (6.6)	3 (1.2)	
Don't know	1	0	
Missing	9	0	
**Vegetable consumption**			0.101
Daily	202 (82.1)	225 (88.6)	
Weekly	40 (16.3)	28 (11.0)	
Monthly	1 (0.4)	1 (0.4)	
Never	3 (1.2)	0 (0.0)	
Don't know	0	0	
Missing	8	0	
**Fiber-rich vegetable consumption**			<0.001
Daily	22 (9.2)	53 (20.9)	
Weekly	161 (67.1)	180 (71.1)	
Monthly	48 (20.0)	19 (7.5)	
Never	9 (3.8)	1 (0.4)	
Don't know	5	0	
Missing	9	1	
**Sugar containing drink consumption**			0.028
Weekly	185 (76.4)	170 (67.2)	
Monthly	20 (8.3)	39 (15.4)	
Never	37 (15.3)	44 (17.4)	
Don't know	3	1	
Missing	9	0	
**Antibiotic use**			<0.001
More than once per month	0 (0.0)	2 (0.8)	
Monthly	11 (4.5)	4 (1.6)	
Seldom	189 (77.5)	120 (47.2)	
Never	44 (18.0)	128 (50.4)	
Missing	10	0	
**Probiotic use**			<0.001
Daily	50 (20.5)	2 (0.8)	
Weekly	56 (23.0)	3 (1.2)	
Monthly	17 (7.0)	4 (1.6)	
Seldom	68 (27.9)	47 (18.5)	
Never	53 (21.7)	198 (78.0)	
Missing	10	0	
**Smoking status**			0.009
Current smoker	53 (22.9)	32 (12.6)	
Past smoker	51 (22.1)	58 (22.8)	
Passive smoker	7 (3.0)	17 (6.7)	
Non-smoker	120 (51.9)	147 (57.9)	
Missing	23	0	

Results of the binary logistic regression are shown in supplementary table S2. A family history of acute appendicitis had the largest contribution to the risk of acute appendicitis. Probiotic use as well as antibiotic use increased the risk, while fruit and fiber-rich vegetable consumption decreased the risk of developing the disease. Having grown up in a rural environment and having a lower education also increased the risk of acute appendicitis.

### 3.2 Complicated versus uncomplicated appendicitis in the Belgian cohort

Characteristics of complicated and uncomplicated appendicitis patients are presented in Table 2. Complicated appendicitis patients were on average older (37.3 versus 30.8 years), more often lived in rural areas at the time of appendicitis (50.6 versus 35.7%) and had more contact with farm animals during their youth. Complicated appendicitis patients also waited significantly longer before going to the hospital, and had a longer length of hospitalization (average of 5.3 and 2.6 days respectively).

**Table 2:** Characteristics of patients with complicated and uncomplicated appendicitis in the Belgian population.

Demographics	Complicated (n = 114)	Uncomplicated (n = 260)	p-value
	n (%)	n (%)	
**Gender**			0.052
Male	69 (60.5)	129 (49.6)	
Female	45 (39.5)	131 (50.4)	
Missing	0	0	
Age, Mean ± SD (Range)	37.3 ± 19.3 (5–79)	30.8 ± 16.8 (5–81)	0.002
**Ethnicity**			0.712
European	107 (98.2)	241 (96.0)	
North African	1 (0.9)	6 (2.4)	
Subsaharan African	0 (0.0)	1 (0.4)	
Asian	1 (0.9)	3 (1.2)	
Missing	5	9	
**Marital State**			0.544
Single	12 (11.4)	23 (9.3)	
Living with partner, family or community	93 (88.6)	224 (90.7)	
Missing	9	13	
**Living Area**			0.009
Rural	55 (50.6)	89 (35.7)	
(Sub)urban	54 (49.5)	160 (64.3)	
Missing	5	11	
**Education**			0.149
Primary school	7 (6.5)	6 (2.4)	
Secondary school	34 (31.5)	71 (28.2)	
Higher education	38 (35.2)	87 (34.5)	
Studying	29 (26.9)	88 (34.9)	
Missing	6	8	
**Acute appendicitis**			
**Family history of acute appendicitis**			0.300
Yes	64 (61.5)	131 (55.5)	
No	40 (38.5)	105 (44.5)	
Missing	10	24	
Length of stay, Mean ± SD (Range)	5.3 ± 2.9 (2–21)	2.6 ± 1.4 (2–18)	<0.001
**Exposures**			
**Living area in youth**			0.060
Rural	60 (56.1)	113 (45.2)	
Urban	47 (43.9)	137 (54.8)	
Missing	3	10	
**Contact with farm animals during youth**			0.004
Daily to monthly	55 (51.4)	89 (35.3)	
Seldom or never	52 (48.6)	163 (64.7)	
Missing	7	8	
**Living with pets**			0.590
Yes	55 (50.5)	136 (53.5)	
No	54 (49.5)	118 (46.5)	
Missing	5	6	
**Breastfeeding**			0.732
No	28 (31.5)	73 (33.5)	
Yes	61 (68.5)	145 (66.5)	
Don't know	17	32	
Missing	8	10	
**Duration**			0.792
0–3 months	17 (42.5)	39 (38.2)	
3–6 months	11 (27.5)	34 (33.3)	
more than 6 months	12 (30.0)	29 (28.4)	
Don't know	21	40	
Missing	0	3	
**Vegan/vegetarian**			0.163
Yes	1 (0.9)	9 (3.6)	
No	107 (99.1)	244 (96.4)	
Missing	6	7	
**Meat consumption**			0.917
Daily	81 (76.4)	177 (75.0)	
Weekly	23 (21.7)	53 (22.5)	
Monthly	2 (1.9)	6 (2.5)	
Don't know	0	0	
Missing	1	8	
**Fruit consumption**			0.922
Daily	50 (45.9)	111 (44.2)	
Weekly	45 (41.3)	103 (41.0)	
Monthly	8 (7.3)	24 (9.6)	
Never	6 (5.5)	13 (5.2)	
Don't know	0	1	
Missing	5	8	
**Vegetable consumption**			0.114
Daily	99 (90.8)	204 (80.6)	
Weekly	9 (8.3)	44 (17.4)	
Monthly	0 (0.0)	1 (0.4)	
Never	1 (0.9)	4 (1.6)	
Don't know	0	0	
Missing	5	7	
**Fiber-rich vegetable consumption**			0.522
Daily	14 (13.2)	24 (9.7)	
Weekly	73 (68.9)	169 (68.4)	
Monthly	17 (16.0)	43 (17.3)	
Never	2 (1.9)	11 (4.5)	
Don't know	2	5	
Missing	6	8	
**Sugar containing drink consumption**			0.555
Weekly	76 (71.0)	190 (75.7)	
Monthly	13 (12.1)	22 (8.8)	
Never	18 (16.8)	39 (15.5)	
Don't know	1	2	
Missing	6	7	
**Antibiotic use**			0.750
More than once per month	0 (0.0)	1 (0.4)	
Monthly	3 (2.8)	12 (4.8)	
Seldom	86 (79.6)	196 (78.1)	
Never	19 (17.6)	42 (16.7)	
Missing	6	9	
**Probiotic use**			0.518
Daily	25 (22.9)	50 (19.9)	
Weekly	22 (20.2)	51 (20.3)	
Monthly	5 (4.6)	25 (10.0)	
Seldom	30 (27.5)	70 (27.9)	
Never	27 (24.8)	55 (21.9)	
Missing	6	9	
**Smoke status**			0.321
Current smoker	22 (23.7)	44 (21.6)	
Past smoker	27 (29.0)	44 (21.6)	
Passive smoker	3 (3.2)	4 (2.0)	
Non-smoker	41 (44.1)	112 (54.9)	
Missing	21	54	
**Delay to diagnosis (hours)**			0.008
0-24	17 (15.6)	65 (25.7)	
24-48	35 (32.1)	98 (38.7)	
>48	57 (52.3)	90 (35.6)	
Missing	7	5	

The results of the binary logistic regression are shown in supplementary table S3. In the final model, being male, having a higher age, and a delay to diagnosis of more than 48 h were risk factors for developing complicated acute appendicitis. Environmental factors including lifestyle were not significant in the development of complicated acute appendicitis.

### 3.3 Gangrenous versus non-gangrenous appendicitis in the Belgian cohort

Characteristics of non-gangrenous and gangrenous appendicitis patients are shown in Table 3. Patients with gangrenous appendicitis tended to be older (37.6 versus 30.6 years). Their length of stay was also longer (average of 4.1 and 3.1 days respectively). In contrast to complicated appendicitis, delay was not associated with gangrenous appendicitis.

**Table 3:** Characteristics of patients with gangrenous and non-gangrenous appendicitis in the Belgian population.

Demographics	Gangrenous (n = 116)	Non-gangrenous (n = 258)	p-value
	n (%)	n (%)	
**Gender**			0.211
Male	67 (57.8)	131 (50.8)	
Female	49 (42.2)	127 (49.2)	
Missing	0	0	
Age, Mean ± SD (Range)	37.6 ± 20.3 (5–81)	30.6 ± 16.2 (5–75)	0.001
**Ethnicity**			0.502
European	111 (98.2)	237 (96.0)	
North African	2 (1.8)	5 (2.0)	
Subsaharan African	0 (0.0)	1 (0.4)	
Asian	0 (0.0)	4 (1.6)	
Missing	3	11	
**Marital Status**			0.950
Single	11 (10.1)	24 (9.9)	
Living with partner, family or community	98 (89.9)	219 (90.1)	
Missing	7	15	
**Living Area**			0.292
Rural	50 (44.2)	94 (38.4)	
(Sub)urban	63 (55.8)	151 (61.6)	
Missing	3	13	
**Education**			0.551
Primary school	6 (5.3)	7 (2.8)	
Secondary school	35 (31.0)	70 (28.3)	
Higher education	38 (33.6)	87 (35.2)	
Studying	33 (29.5)	84 (33.9)	
Missing	4	10	
**Acute appendicitis**			
**Family history of acute appendicitis**			0.509
Yes	63 (60.0)	132 (56.2)	
No	42 (40.0)	103 (43.8)	
Missing	11	23	
Length of stay (Days), Mean ± SD (Range)	4.1 ± 2.9 (2–21)	3.1 ± 1.8 (2–18)	0.002
**Exposures**			
**Living area in youth**			0.233
Rural	60 (53.1)	113 (46.3)	
Urban	53 (46.9)	131 (53.7)	
Missing	3	14	
**Contact with farm animals during youth**			0.223
Daily to monthly	51 (44.7)	93 (38.0)	
Seldom or never	63 (55.3)	152 (62.0)	
Missing	2	13	
**Living with pets**			0.114
Yes	53 (46.5)	138 (55.4)	
No	61 (53.5)	111 (44.6)	
Missing	2	9	
**Breastfeeding**			0.194
No	26 (27.7)	75 (35.2)	
Yes	68 (72.3)	138 (64.8)	
Don't know	19	30	
Missing	3	15	
**Duration**			0.437
0–3 months	14 (31.8)	42 (42.9)	
3–6 months	15 (34.1)	30 (30.6)	
more than 6 months	15 (34.1)	26 (26.5)	
Don't know	23	38	
Missing	1	2	
**Vegan/vegetarian**			0.137
Yes	1 (0.9)	9 (3.6)	
No	113 (99.1)	238 (96.4)	
Missing	2	11	
**Meat consumption**			0.852
Daily	81 (73.6)	177 (76.3)	
Weekly	26 (23.6)	50 (21.6)	
Monthly	3 (2.7)	5 (2.2)	
Don't know	0	0	
Missing	3	6	
**Fruit consumption**			0.018
Daily	63 (55.3)	98 (39.7)	
Weekly	39 (34.2)	119 (44.1)	
Monthly	5 (4.4)	27 (10.9)	
Never	7 (6.1)	12 (4.9)	
Don't know	0	1	
Missing	2	10	
**Vegetable consumption**			0.294
Daily	100 (87.7)	203 (81.9)	
Weekly	14 (12.3)	39 (15.7)	
Monthly	0 (0.0)	1 (0.4)	
Never	0 (0.0)	5 (2.0)	
Don't know	0	0	
Missing	2	10	
**Fiber-rich vegetable consumption**			0.603
Daily	9 (8.0)	29 (12.0)	
Weekly	80 (71.4)	162 (67.2)	
Monthly	20 (17.9)	40 (16.6)	
Never	3 (2.7)	10 (4.1)	
Don't know	1	6	
Missing	3	11	
**Sugar containing drink consumption**			0.291
Weekly	79 (69.9)	187 (76.3)	
Monthly	11 (9.7)	24 (9.8)	
Never	23 (20.4)	34 (13.9)	
Don't know	0	3	
Missing	3	10	
**Antibiotic use**			0.800
More than once per month	0 (0.0)	1 (0.4)	
Monthly	5 (4.4)	10 (4.1)	
Seldom	92 (80.7)	190 (77.6)	
Never	17 (14.9)	44 (18.0)	
Missing	2	13	
**Probiotic use**			0.273
Daily	28 (24.6)	47 (19.1)	
Weekly	27 (23.7)	46 (18.7)	
Monthly	7 (6.1)	23 (9.3)	
Seldom	25 (21.9)	75 (30.5)	
Never	27 (23.7)	55 (22.4)	
Missing	2	12	
**Smoking status**			0.764
Current smoker	19 (20.2)	47 (23.2)	
Past smoker	26 (27.7)	45 (22.2)	
Passive smoker	2 (2.1)	5 (2.5)	
Non-smoker	47 (50.0)	106 (52.2)	
Missing	22	55	
**Delay to diagnosis (hours)**			0.624
0-24	22 (29.6)	60 (24.0)	
24-48	44 (39.3)	89 (35.6)	
>48	46 (41.1)	101 (40.4)	
Missing	4	8	

Results from the binary logistic regression analysis are shown in supplementary table S4. In the final model, only high age remained as a significant factor in the development of gangrenous acute appendicitis, and no differences in environmental factors, including lifestyle, were of importance in the development of gangrenous acute appendicitis.

### 3.4 Patient and control characteristics within the second cohort (NBS)

Significantly more individuals with a history of appendectomy were female (57.5%) and the average age was higher (61.4 versus 52.3 years, data not shown).

Characteristics of individuals with and without a history of appendectomy from the NBS population after matching for age and gender are shown in Table 4. Individuals with a history of appendectomy had on average a lower level of education. Both fruit and vegetable consumption were lower in individuals with a history of appendectomy. More individuals with a history of appendectomy were past or current smokers.

**Table 4:** Characteristics of individuals with a history of appendectomy and those without, the NBS population matched 1:2 for age and gender.

Demographics	Appendectomy (n = 1063)	No appendectomy (n = 2126)	p-value
	n (%)	n (%)	
**Gender**			1.000
Male	452 (42.5)	904 (42.5)	
Female	611 (57.5)	1222 (57.5)	
Age, mean ± SD (Range)	61.4 ± 16.7 (18–95)	60.5 ± 17.5 (18–96)	0.151
**Marital Status**			0.088
Single	327 (30.8)	718 (33.8)	
Living with partner, family or community	734 (69.2)	1404 (66.2)	
Missing	2	4	
**Education**			0.005
Primary school	325 (30.9)	596 (28.2)	
Secondary school	325 (30.9)	579 (27.4)	
Higher education	403 (38.3)	936 (44.3)	
Missing	10	15	
Appendectomy at age, mean ± SD (Range)	21.7 ± 13.1 (1–80)	n.a	n.a
**Exposures**			
**Fruit consumption**			0.019
Never	45 (4.3)	68 (3.3)	
1–2 days per week	193 (18.4)	308 (14.7)	
3–5 days per week	182 (17.3)	371 (17.8)	
(Almost) daily	630 (60.0)	1343 (64.3)	
Missing	13	36	
**Vegetable consumption**			0.043
Never	2 (0.2)	6 (0.3)	
1–2 days per week	35 (3.4)	49 (2.3)	
3–5 days per week	221 (21.2)	376 (18.0)	
(Almost) daily	785 (75.3)	1659 (79.4)	
Missing	20	36	
**Whole-wheat product consumption**			0.841
Never	50 (4.8)	92 (4.4)	
1–2 days per week	48 (4.6)	91 (4.4)	
3–5 days per week	93 (8.9)	172 (8.2)	
(Almost) daily	851 (81.7)	1730 (83.0)	
Missing	21	41	
**Meat consumption**			0.763
Never	30 (2.9)	67 (3.2)	
1–2 days per week	93 (8.9)	198 (9.5)	
3–5 days per week	297 (28.4)	608 (29.2)	
(Almost) daily	626 (59.8)	1206 (58.0)	
Missing	17	47	
**Smoking status**			0.019
Current smoker	255 (24.2)	444 (21.0)	
Past smoker	476 (45.2)	929 (43.9)	
Non-smoker	321 (30.5)	741 (35.1)	
Missing	11	12	

The results from the binary logistic regression are shown in supplementary table S5. In this population, individuals with a history of appendectomy more often lived with partner, family or community, had a lower level of education, and ate less fruit.

### 3.5 Comparison of characteristics between the Belgian acute appendicitis population and NBS individuals with a history of appendectomy, and the control population (500FG) and NBS individuals without a history of appendectomy

In order to ensure that potential differences between the Belgian appendicitis cohort and the 500FG control population represented differences between patients and controls, rather than between Belgian and Dutch individuals, a comparison was made between the patients from the Belgian acute appendicitis population and individuals with a history of appendectomy, maximum 15 years before questioning, from the Dutch NBS population. Characteristics of both populations are summarized in supplementary table S6. The mean age of the NBS patients at the time of appendicitis was 22, which is lower than the mean age of the Belgian patients (33 years). Populations were matched for age (age at appendicitis in case of patients) and gender.

Belgian patients consumed more vegetables as well as meat, more often lived with their family or in communities, and smoked less. Controls from the 500FG cohort also more often lived with family or in communities, smoked less, consumed more fruit and vegetables and less meat.

Both populations differed regarding fruit and vegetable consumption and meat (500FG individuals consumed more). Controls from the 500FG population also more often lived with family or in communities, and smoked less.

## 4 Discussion

The analysis of exposures in the acute appendicitis cohort compared to 500FG controls shows that a number of environmental and lifestyle factors are associated with the occurrence of appendicitis. Overall, appendicitis was associated with a less healthy diet.

Diets rich in sugar and low in fruit and vegetables seem to be associated with appendicitis risk. Low fiber diets have previously been shown to be associated with a higher risk of acute appendicitis [4], which is confirmed in this study, as well as the effect of smoking [8]. Breastfeeding and childhood environment are associated with appendicitis, as patients were less often breastfed and more often grew up in rural environments. Both factors can affect the development of the immune system [16,17]. A positive family history was one of the most important factors in this study, which confirms earlier reports [18]. More than half of the patients from the Belgian acute appendicitis cohort indicated a positive family history, strongly suggesting that apart from dietary habits and general lifestyle, genetic factors are at play as well. The considerably low number of individuals with a positive family history in the 500FG cohort, is likely due to information bias, as healthy control individuals might inquire less about family history than patients that are ill at the time of questioning.

Although the influence of dietary habits on the risk of appendicitis was confirmed in the NBS population, it was less apparent. The effects of smoking were confirmed, as individuals with a history of appendectomy were more often smokers or past smokers. While differences in level of education between the Belgian appendicitis cohort and the 500FG cohort were likely a consequence of the 500FG population mostly consisting of university students, this finding could be replicated in the population based NBS cohort as well. Level of education is seen as an important indicator of socioeconomic status and a higher educational level has previously been shown to be associated with lower incidences of appendicitis [19]. Differences in marital status and current environment found between the Belgian appendicitis cohort and the 500FG cohort could not be replicated in the NBS population, leading to the conclusion that these findings are likely a consequence of the choice of the control population.

When comparing the individuals with a history of appendectomy from the NBS with the Belgian acute appendicitis patients, as well as controls from the NBS with the 500FG population, lifestyle and environmental factors differed significantly. This may be a consequence of different recruitment periods and methods, raising the question of whether the differences in lifestyle and environment found between the acute appendicitis and control population could be attributed to differences between Belgian and Dutch individuals. Although patients from both populations were recruited in border areas only 100 km apart, and during the same period, cultural differences between Belgian and Dutch individuals also need to be taken into account [20].

Differences in characteristics within patient and control populations may also be a result of different questionnaires, as not all variables were collected in both questionnaires and for some variables, data needed to be transformed in order to be able to compare these populations. The long time-interval between the occurrence of appendectomy and questioning in the NBS population most likely also influenced the results.

Importantly, while appendicitis patients from the Belgian cohort were more often male, individuals with a history of appendectomy from the NBS population were more often female. Negative appendectomy rates are higher in women than in men, mainly due to misdiagnosed gynecologic conditions [21]. Due to better diagnostics, the negative appendectomy rate has decreased over the past years, a trend which is most pronounced in women [22]. This can indicate that not all individuals with a history of appendectomy in the NBS population were true appendicitis cases.

Not surprisingly, within the Belgian patient population, the severity of acute appendicitis influenced the outcome of treatment, as patients with more severe appendicitis had a longer length of stay. Complicated appendicitis diagnosed by the surgeon was furthermore associated with a longer delay to diagnosis, which confirms findings from previous studies [23,24]. Interestingly, the histological finding of gangrenous appendicitis was not associated with delay. In addition, the observation that only approximately half of gangrenous appendicitis cases and complicated appendicitis cases overlapped suggests that a histologically gangrenous or necrotic appendix is not always accompanied by macroscopic perforation, peri-appendicular abscess or peritonitis, and severe macroscopic peri appendicular inflammation in turn is not always associated with local tissue necrosis in the appendix. The distinction between both types of severe appendicitis has been illustrated in previous research as well, as for example, cytokine profiles appear to differ between complicated or phlegmonous appendicitis, and gangrenous appendicitis [[25], [26], [27]]. This distinction should further be taken into account in future studies into factors contributing to severity of acute appendicitis, in order to clarify the underlying processes.

A limitation of this study is that characteristics of the Belgian acute appendicitis patients could not be compared to Belgian controls. The requirement for blood and fecal samples from a control population to address other research questions of the HAPPIEST study were met by insurmountable recruitment problems. Even though the 500FG population was recruited in a geographical area bordering the HAPPIEST cohort area, certain cultural differences between Belgians and Dutch people [20], as well as the fact that this population consisted mostly of university students, do call for caution when interpreting these results.

In conclusion, this study confirms that lifestyle and environment may affect the risk of developing acute appendicitis, and to a lesser extent the severity of the disease. It may be beneficial to take these factors into account in future research into the etiology of acute appendicitis.

## Ethical statement

All patients and healthy controls included in this study, or their parents or guardians, gave written informed consent before participation. The study was approved by the Medical Ethics Committee of Jessa Hospital, Hasselt, Belgium. The HAPPIEST study was registered at Clinicaltrials.gov, Identifier NCT02391675.

The 500FG study was approved by the Ethical Committee of Radboud University Nijmegen (NL42561.091.12, 2012/550). All volunteers gave written informed consent before any material was collected.

The NBS was approved by the Radboud university medical center Institutional Review Board. All participants gave written informed consent.

## Author contribution statement

Toon Peeters: Conceived and designed the experiments; Performed the experiments; Analyzed and interpreted the data; Contributed reagents, materials, analysis tools or data; Wrote the paper.

Bert Houben; Peter Cools; Valentino D'Onofrio; Sandrina Martens: Performed the experiments; Contributed reagents, materials, analysis tools or data.

Yati Thys: Conceived and designed the experiments; Performed the experiments; Contributed reagents, materials, analysis tools or data.

Martin Jaeger; Marije Doppenberg-Oosting: Contributed reagents, materials, analysis tools or data.

Mihai G Netea; Inge C Gyssens: Conceived and designed the experiments; Analyzed and interpreted the data; Wrote the paper.

## Funding statement

This study is part of the Limburg Clinical Research Program (LCRP) UHasselt-ZOL-Jessa, supported by the foundation Limburg Sterk Merk, province of Limburg, Flemish government, 10.13039/501100009550Hasselt University, Ziekenhuis Oost-Limburg and Jessa Hospital.

## Data availability statement

Data will be made available on request.

## Declaration of interest's statement

The authors declare no conflict of interest.

## References

[1] Stewart B., Khanduri P., McCord C., Ohene-Yeboah M., Uranues S., Vega R.F. (2014). Global disease burden of conditions requiring emergency surgery. Br. J. Surg..

[2] Williams J.G., Roberts S.E., Ali M.F., Cheung W.Y., Cohen D.R., Demery G. (2007). Gastroenterology services in the UK. The burden of disease, and the organisation and delivery of services for gastrointestinal and liver disorders: a review of the evidence. Gut.

[3] Lee J.H., Park Y.S., Choi J.S. (2010). The epidemiology of appendicitis and appendectomy in South Korea: national registry data. J. Epidemiol..

[4] Burkitt D.P. (1971). The aetiology of appendicitis. Br. J. Surg..

[5] Barker D.J. (1985). Acute appendicitis and dietary fibre: an alternative hypothesis. Br. Med. J..

[6] Barker D.J., Osmond C., Golding J., Wadsworth M.E. (1988). Acute appendicitis and bathrooms in three samples of British children. Br. Med. J..

[7] Barker D.J., Morris J.A., Simmonds S.J., Oliver R.H. (1988). Appendicitis epidemic following introduction of piped water to Anglesey. J. Epidemiol. Community Health.

[8] Oldmeadow C., Wood I., Mengersen K., Visscher P.M., Martin N.G., Duffy D.L. (2008). Investigation of the relationship between smoking and appendicitis in Australian twins. Ann. Epidemiol..

[9] Lin K.B., Lai K.R., Yang N.P., Chan C.L., Liu Y.H., Pan R.H. (2015). Epidemiology and socioeconomic features of appendicitis in Taiwan: a 12-year population-based study. World J. Emerg. Surg..

[10] Lin S., Sun M., Fitzgerald E., Hwang S.A. (2016). Did summer weather factors affect gastrointestinal infection hospitalizations in New York State?. Sci. Total Environ..

[11] Ferris M., Quan S., Kaplan B.S., Molodecky N., Ball C.G., Chernoff G.W. (2017). The global incidence of appendicitis: a systematic review of population-based studies. Ann. Surg..

[12] Schirmer M., Smeekens S.P., Vlamakis H., Jaeger M., Oosting M., Franzosa E.A. (2016). Linking the human gut microbiome to inflammatory cytokine production capacity. Cell.

[13] Galesloot T.E., Vermeulen S.H., Swinkels D.W., de Vegt F., Franke B., den Heijer M. (2017). Cohort profile: the nijmegen biomedical study (NBS). Int. J. Epidemiol..

[14] Davey G.K., Spencer E.A., Appleby P.N., Allen N.E., Knox K.H., Key T.J. (2003). EPIC-Oxford: lifestyle characteristics and nutrient intakes in a cohort of 33 883 meat-eaters and 31 546 non meat-eaters in the UK. Publ. Health Nutr..

[15] Crowe F.L., Appleby P.N., Allen N.E., Key T.J. (2011). Diet and risk of diverticular disease in Oxford cohort of European Prospective Investigation into Cancer and Nutrition (EPIC): prospective study of British vegetarians and non-vegetarians. BMJ.

[16] Laouar A. (2020). Maternal leukocytes and infant immune programming during breastfeeding. Trends Immunol..

[17] Kilpeläinen M., Terho E.O., Helenius H., Koskenvuo M. (2002). Childhood farm environment and asthma and sensitization in young adulthood. Allergy.

[18] Li H.M., Yeh L.R., Huang Y.K., Hsieh M.Y., Yu K.H., Kuo C.F. (2018). Familial risk of appendicitis: a nationwide population study. J. Pediatr..

[19] Golz R.A., Flum D.R., Sanchez S.E., Liu X., Donovan C., Drake F.T. (2020). Geographic association between incidence of acute appendicitis and socioeconomic status. JAMA Surg..

[20] Meeuwesen L., van den Brink-Muinen A., Hofstede G. (2009). Can dimensions of national culture predict cross-national differences in medical communication?. Patient Educ. Counsel..

[21] Seetahal S.A., Bolorunduro O.B., Sookdeo T.C., Oyetunji T.A., Greene W.R., Frederick W. (2011). Negative appendectomy: a 10-year review of a nationally representative sample. Am. J. Surg..

[22] Raja A.S., Wright C., Sodickson A.D., Zane R.D., Schiff G.D., Hanson R. (2010). Negative appendectomy rate in the era of CT: an 18-year perspective. Radiology.

[23] Omundsen M., Dennett E. (2006). Delay to appendicectomy and associated morbidity: a retrospective review. ANZ J. Surg..

[24] Saar S., Talving P., Laos J., Podramagi T., Sokirjanski M., Lustenberger T. (2016).

[25] Peeters T., Martens S., D'Onofrio V., Stappers M.H.T., van der Hilst J.C.H., Houben B. (2020). An observational study of innate immune responses in patients with acute appendicitis. Sci. Rep..

[26] Ruber M., Andersson M., Petersson B.F., Olaison G., Andersson R.E., Ekerfelt C. (2010). Systemic Th17-like cytokine pattern in gangrenous appendicitis but not in phlegmonous appendicitis. Surgery.

[27] Ruber M., Berg A., Ekerfelt C., Olaison G., Andersson R.E. (2006). Different cytokine profiles in patients with a history of gangrenous or phlegmonous appendicitis. Clin. Exp. Immunol..

