# Supplementary material for: An observational study on lifestyle and environmental risk factors in patients with acute appendicitis
Source: Heliyon. 2023 Apr 1;9(4):e15131. doi: 10.1016/j.heliyon.2023.e15131 (PMC10147974; doi:10.1016/j.heliyon.2023.e15131)
Supplement: Multimedia component 1 [file mmc1.pdf]

An observational study on lifestyle and environmental risk factors in patients with acute appendicitis

Toon Peeters*[1,2,3,4], Bert Houben[5], Peter Cools[6], Yati Thys[1,2], Valentino D'Onofrio[1,2,3,4], Sandrina Martens[2,8], Martin Jaeger[3,4,7], Marije Doppenberg-Oosting[3], Mihai G. Netea[3,4], Inge C. Gyssens[1,2,3,4]

1. Department of Infectious Diseases and Immunity, Jessa Hospital, 3500 Hasselt, Belgium.
2. Faculty of Medicine and Life Sciences, Hasselt University, 3500 Hasselt, Belgium.
3. Department of Internal Medicine, Radboud University Medical Center, 6525 GA Nijmegen, The Netherlands.
4. Radboudumc Center for Infectious Diseases (RCI), Radboud University Medical Center, Nijmegen, The Netherlands
5. Department of Abdominal and Oncological Surgery, Jessa Hospital, 3500 Hasselt, Belgium.
6. Department of Abdominal Surgery, GZA Hospital, Antwerp, Belgium.
7. Radboud Institute for Molecular Life Sciences, Radboud University Medical Center, Nijmegen, the Netherlands
8. Current affiliation: Department of Experimental Pathology, Vrije Universiteit Brussel (VUB), 1090 Brussels, Belgium.

Corresponding author:

Toon Peeters

Hasselt University, Campus Diepenbeek

Agoralaan Gebouw C

BE 3590 Diepenbeek

e-mail: toon.peeters@uhasselt.be

Supplementary table S1. Comparison of questionnaires used in the Belgian acute appendicitis and 500FG population versus the NBS population, and transformation of the data for comparing the Belgian patients and 500FG controls with individuals with and without a history of appendectomy from the NBS population, respectively.

Variable	Options		
	HAPPIEST - 500FG	NBS	Combined
Fruit consumption			
	Never	Never	Never/monthly
	Monthly		
	Weekly	1-2 days per week	Weekly
		3-5 days per week	
	Dayly	(Almost) Dayly	(Almost) Dayly
Vegetable consumption			
	Never	Never	Never/monthly
	Monthly		
	Weekly	1-2 days per week	Weekly
		3-5 days per week	
	Dayly	(Almost) Dayly	(Almost) Dayly
Meat consumption			
	Vegan/vegetarian	Never	Never/monthly
	Monthly		
	Weekly	1-2 days per week	Weekly
		3-5 days per week	
	Daily	(Almost) Daily	(Almost) Daily
Smoking status			
	Current smoker	Current smoker	Current smoker
	Past smoker	Past smoker	Past smoker
	Non-smoker	Non-smoker	Non-smoker

Supplementary table S2. Summary of binary logistic regression analysis on the Belgian acute appendicitis population and 500FG population, predicting the risk of acute appendicitis.

Variable	p-value	OR	95% CI	
			Lower	Upper
Living Area				
Rural	< 0.001	6.326	2.680	14.931
Education	< 0.001			
Primary/secondary school	< 0.001	7.447	2.822	19.650
Higher education	0.075	1.995	0.932	4.274
Family history of acute appendicitis				
Yes	< 0.001	21.703	8.850	53.223
Fruit consumption	< 0.001			
Weekly	0.013	2.437	1.204	4.932
Monthly to never	< 0.001	9.021	3.041	26.760
Fiber-rich vegetable consumption	0.006			
Weekly	0.067	2.359	0.942	5.910
Monthly to never	0.001	6.239	2.028	19.195
Antibiotic use	< 0.001			
Once or more per month	0.530	1.756	0.304	10.154
Seldom	< 0.001	4.987	2.400	10.363
Probiotic use				
Daily to rarely	< 0.001	16.924	8.168	35.070

Supplementary table S3. Summary of binary logistic regression analysis on the Belgian acute appendicitis population, predicting the risk of complicated acute appendicitis.

Variable	p-value	OR	95% CI	
			Lower	Upper
Gender				
Male	0.030	1.691	1.053	2.716
Age	0.001	1.022	1.008	1.035
Delay to Diagnosis*	0.016			
0-24h	0.008	0.419	0.221	0.796
24-48h	0.047	0.591	0.351	0.994

* Reference > 48h

Supplementary table S4. Summary of binary logistic regression analysis on the Belgian acute appendicitis population, predicting the risk of gangrenous acute appendicitis.

Variable	p-value	OR	95% CI	
			Lower	Upper
Age	0.001	1.022	1.009	1.035

Supplementary table S5. Summary of binary logistic regression analysis on the NBS population, predicting the risk of having had an appendectomy.

Variable	p-value	OR	95% CI	
			Lower	Upper
Marital state				
Single	0.048	0.848	0.720	0.999
Education	0.007			
Primary school	0.010	1.271	1.060	1.523
Secondary school	0.006	1.287	1.074	1.543
Vegetable consumption	0.017			
Never	0.959	0.957	0.184	4.969
1-2 days per week	0.047	1.583	1.006	2.492
3-5 days per week	0.007	1.295	1.072	1.564

Supplementary table S6. Characteristics of patient and control populations, matched for gender and age (fuzz factor of 5 years). Patients from the NBS population were selected for having had appendicitis maximum 15 years before questioning. Patients from the Belgian acute appendicitis and NBS populations were matched 2:1 for gender and age at which they had an appendectomy, controls from the 500FG and NBS populations were matched 1:3 for gender and current age. p-value1 indicates the comparison between the patient populations, p-value2 indicates the comparison between the control populations.

	Patients HAPPIEST (n=210)	Patients NBS (n=105)	p-value[1]	Controls 500FG (n=321)	Controls NBS (n=963)	p-value[2]
Demographics						
Gender			1.000			1.000
Male	82 (39.0)	41 (39.0)		138 (43.0)	414 (43.0)	
Female	128 (61.0)	64 (61.0)		183 (57.0)	549 (57.0)	
Age, mean ± SD (Range)	34.19 ± 19.02 (5-81)	41.25 ± 18.09 (18-87)	0.002	32.10 ± 15.31 (20-73)	34.10 ± 14.68 (18-78)	0.037
Marital Status			< 0.001			< 0.001
Single	22 (11.1)	39 (37.1)		83 (25.9)	384 (39.9)	
Living with partner, family or community	176 (88.9)	66 (62.9)		238 (74.1)	578 (60.1)	
Missing	12	0		0	1	
Age at appendectomy, mean ± SD (Range)	34.19 ± 19.02 (5-81)	34.35 ± 18.88 (7-80)	0.941	n.a	n.a	n.a
Exposures						
Fruit consumption			0.518			< 0.001
Never/monthly	23 (11.4)	8 (7.6)		13 (4.0)	57 (6.0)	
Weekly	83 (41.3)	48 (45.7)		92 (28.7)	484 (50.6)	
(Almost) Daily	95 (47.3)	49 (46.7)		216 (67.6)	416 (43.5)	
Missing	9	0		0	6	
Vegetable consumption			0.001			< 0.001
Never/monthly	2 (1.0)	0 (0.0)		1 (0.3)	5 (0.5)	
Weekly	22 (10.9)	28 (26.9)		29 (9.0)	317 (33.1)	
(Almost) Dayly	178 (88.1)	76 (73.1)		291 (90.7)	635 (66.4)	
Missing	8	1		0	6	
Meat consumption			0.047			< 0.001
Never/monthly	11 (5.6)	3 (2.9)		34 (11.7)	50 (5.2)	

Weekly	48 (24.2)	39 (37.1)		87 (30.0)	400 (41.8)	
(Almost) Daily	139 (70.2)	63 (60.0)		169 (58.3)	508 (53.0)	
Missing	12	0		31	5	
Smoking status			0.001			< 0.001
Current smoker	37 (22.6)	28 (26.7)		48 (15.1)	247 (25.7)	
Past smoker	31 (18.9)	39 (37.1)		62 (19.5)	265 (27.6)	
Non-smoker	96 (58.5)	38 (36.2)		208 (65.4)	449 (46.7)	
Missing	46	0		3	2	
