# Supplementary material for: An observational study on lifestyle and environmental risk factors in patients with acute appendicitis
Source: Heliyon. 2023 Apr 1;9(4):e15131. doi: 10.1016/j.heliyon.2023.e15131 (PMC10147974; doi:10.1016/j.heliyon.2023.e15131)
Supplement: Multimedia component 2 [file mmc2.doc]

| **HAPPIEST cohort Studie Vragenlijst: LIFESTYLE Pv1 (≥ 15 t.e.m. 85 jaar)** |
| --- |

Geachte Mijnheer / Mevrouw,

U doet mee aan de **‘HAPPIEST studie’**, een studie naar de risicofactoren van acute appendicitis en de daarbij mogelijk optredende complicaties. In de informatiebrochure die u reeds van de behandelende arts heeft ontvangen, vindt u meer gedetailleerde informatie over de studie.

Wij vragen patiënten die meedoen aan de studie deze vragenlijst in te vullen. Wij vragen u daarbij steeds om **uw eigen mening**. Er zijn geen goede of foute antwoorden. Wij zullen zorgvuldig met deze vertrouwelijke informatie omgaan. Het invullen duurt ongeveer **10-15 minuten**.

De ingevulde **vragenlijst** dient u **voor het verlaten van het ziekenhuis te deponeren in de daarvoor bestemde box op uw afdeling**. Als u vragen heeft over deze vragenlijst, aarzel dan niet om contact met ons op te nemen (zie contactpersonen, bijlage patiënt informatiebrochure).

Wij willen u alvast hartelijk bedanken voor het invullen van deze vragenlijst en uw deelname aan de **HAPPIEST studie**!

Met vriendelijke groet,

Namens het ‘HAPPIEST studie’ team

Prof. Dr. I.C. Gyssens, internist-infectioloog / HAPPIEST Prinicipal investigator (PI)

[i.gyssens@aig.umcn.nl](mailto:i.gyssens@aig.umcn.nl) | [inge.gyssens@jessazh.be](mailto:inge.gyssens@jessazh.be) | +32 11 30 94 85

Jessa Ziekenhuis – Campus Virga Jesse

Stadsomvaart 11

3500 Hasselt

België

| **HAPPIEST cohort Studie Vragenlijst: LIFESTYLE Pv1 (≥15 t.e.m. 85 jaar)** |
| --- |

| Datum: ………/………/………  Naam: …......................................................  Voornaam: …......................................................  Geboortedatum: ………/………/………  Telefoon/GSM nummer:…......................................................  E-mailadres: …......................................................  Studienummer: …............................... (in te vullen door onderzoeker/studiemedewerker) |
| --- |

| **SECTIE A : Demografische gegevens**  In deze sectie willen wij vragen **uzelf te beschrijven** aan de hand van een paar vragen.  Al de informatie die wij van u ontvangen zal vertrouwelijk blijven en dusdanig behandeld worden. |
| --- |

**DEM 1** Wat is uw geslacht?

- - Man
  - Vrouw

**DEM 2** Wat is uw leeftijd?

- - 5 – 14 jaar
  - 15 – 24 jaar
  - 25 – 44 jaar
  - 45 – 64 jaar
  - 65+

**DEM 3** Wat is uw huidige burgerlijke staat? (Gelieve één te kiezen die het beste uw huidige situatie beschrijft)

- - Alleen wonend
  - Samenwonend met partner of familieli(e)d(en)
  - Wonend in gemeenschap (studentenkot, rusthuis, instelling, …)

**DEM 4**  Hoeveel kinderen heeft u en wat is hun leeftijd?

1)…………………………………………………………………………………………………………………

2)…………………………………………………………………………………………………………………

3)…………………………………………………………………………………………………………………

4)…………………………………………………………………………………………………………………

5)…………………………………………………………………………………………………………………

6)…………………………………………………………………………………………………………………

7)…………………………………………………………………………………………………………………

**DEM 5** Wat is het hoogste diploma dat u, tot nu toe heeft behaald?

(Opgelet: indien u nog naar school gaat, kleurt u het laatste bolletje)

- - Lager onderwijs
  - Middelbaar onderwijs
  - Hogeschool opleiding
  - Universitaire opleiding
  - Ik ben nog in opleiding (Specifiek:………………………………………………………..)

**DEM 6** Wat is uw huidige werkstatus? (Gelieve één status te kiezen die het beste uw huidige situatie beschrijft)

- - Voltijds/halftijds aan het werken
  - Geen werk, maar op zoek
  - Huisvrouw/huisman
  - Student
  - Op pensioen
  - Anderen (Specifiek:………………………………………………………………………………)

**DEM 7** Wat is uw etnische achtergrond?

- - Europees
  - Noord-Afrika
  - Sub-Saharan Afrika
  - Aziatisch

**DEM 8** Hoe kan u het gebied omschrijven waar u nu woont?

- - Platteland (landelijk)
  - Stad en/of dorpskern (verstedelijkt)

**DEM 9**  Hoe kan u het gebied omschrijven waar u in uw jeugd heeft gewoond?

- - Platteland (landelijk) (Ga naar **DEM 10**)
  - Stad en/of dorpskern (verstedelijkt)

**DEM 10** Heeft u in uw jeugd op een boerderij / hoeve gewoond?

- - Ja
  - Neen

**DEM 11** Kwam u in uw jeugd vaak in contact met dieren (vee: varkens, koeien, schapen, geiten, paarden, kippen, etc.)

- - Dagelijks
  - 1 x per maand
  - Zelden
  - Nooit

**DEM 12** Heeft u huisdieren die bij u in huis leven (katten, honden, hamsters, cavia’s, etc.)?

- - Ja
  - Neen

| **SECTIE B1 : Gezondheid**  In deze sectie polsen wij naar **uw algemene gezondheid**. Al de informatie die wij van u ontvangen zal vertrouwelijk blijven en dusdanig behandeld worden. |
| --- |

**GEZ 1** Hoe vindt u uw gezondheid in het algemeen?

- - Uitstekend
  - Zeer goed
  - Goed
  - Redelijk
  - Slecht

**GEZ 2** Hoe vaak had u de afgelopen week klachten over uw lichamelijke gezondheid?

- - Voortdurend
  - Het grootste deel van de tijd
  - Een deel van de tijd
  - Een klein deel van de tijd
  - Geheel niet

**GEZ 3** Hoe vaak had u de afgelopen week klachten over uw mentale gezondheid (zoals stress, angstige en depressieve gevoelens)?

- - Voortdurend
  - Het grootste deel van de tijd
  - Een deel van de tijd
  - Een klein deel van de tijd
  - Geheel niet

**GEZ 4** Hoe vaak heeft uw lichamelijke en/of mentale gezondheid u de afgelopen week belemmerd in het uitvoeren van uw dagelijkse activiteiten, zoals werk, ontspanning, etc.?

- - Voortdurend
  - Het grootste deel van de tijd
  - Een deel van de tijd
  - Een klein deel van de tijd
  - Geheel niet

**GEZ 5**  Hoe vaak heeft u zich de afgelopen week moe of vermoeid gevoeld?

- - Voortdurend
  - Het grootste deel van de tijd
  - Een deel van de tijd
  - Een klein deel van de tijd
  - Geheel niet

| **SECTIE B2 : Algemene gesteldheid van het darmstelsel**  In deze sectie polsen wij naar de **algemene gesteldheid van uw darmstelsel**. Al de informatie die wij van u ontvangen zal vertrouwelijk blijven en dusdanig behandeld worden. |
| --- |

**DAR 1** Hoe vaak heeft u de afgelopen week buikpijn gehad?

- - Voortdurend
  - Het grootste deel van de tijd
  - Een deel van de tijd
  - Een klein deel van de tijd
  - Geheel niet

**DAR 2** Hoe vaak heeft u in de afgelopen week een opgeblazen gevoel (gevoel van teveel lucht in de buik) gehad?

- - Voortdurend
  - Het grootste deel van de tijd
  - Een deel van de tijd
  - Een klein deel van de tijd
  - Geheel niet

**DAR 3** Hoe vaak heeft u in de afgelopen week last gehad van winderigheid?

- - Voortdurend
  - Het grootste deel van de tijd
  - Een deel van de tijd
  - Een klein deel van de tijd
  - Geheel niet

**DAR 4** Hoe vaak heeft u in de afgelopen week last gehad van rommelingen in de buik?

- - Voortdurend
  - Het grootste deel van de tijd
  - Een deel van de tijd
  - Een klein deel van de tijd
  - Geheel niet

**DAR 5** Hoe vaak heeft u er in de afgelopen week last van gehad dat u veelvuldig ontlasting had (vaker naar het toilet gaan dan anders)?

- - Voortdurend
  - Het grootste deel van de tijd
  - Een deel van de tijd
  - Een klein deel van de tijd
  - Geheel niet

**DAR 6** Hoe vaak heeft u in de afgelopen week last gehad van ernstige aandrang tot het krijgen van ontlasting?

- - Voortdurend
  - Het grootste deel van de tijd
  - Een deel van de tijd
  - Een klein deel van de tijd
  - Geheel niet

**DAR 7**  Hoe vaak heeft u in de afgelopen week last gehad van dunnere ontlasting
(diarree)?

- - Voortdurend
  - Het grootste deel van de tijd
  - Een deel van de tijd
  - Een klein deel van de tijd
  - Geheel niet

**DAR 8**  Hoe vaak heeft u in de afgelopen week last gehad van verstopping
(constipatie) ?

- - Voortdurend
  - Het grootste deel van de tijd
  - Een deel van de tijd
  - Een klein deel van de tijd
  - Geheel niet

**DAR 9** Hoe vaak heeft u in de afgelopen week last gehad van misselijkheid?

- - Voortdurend
  - Het grootste deel van de tijd
  - Een deel van de tijd
  - Een klein deel van de tijd
  - Geheel niet

**DAR 12** Wanneer heeft u het eerste duidelijke symptoom van appendicitis (navelpijn, pijn in de rechteronderbuik) ervaren?

- - Vandaag
  - Vannacht
  - Gisteren
  - 2 dagen geleden
  - > 2 dagen geleden

**DAR 13** Wanneer heeft u voor het eerst uw huisarts of een arts geraadpleegd?

- - Vandaag
  - Vannacht
  - Gisteren
  - 2 dagen geleden
  - > 2 dagen geleden

**DAR 14** Zijn er naaste bloedverwanten (1^ste^ graad: vader, moeder, broers en zussen; 2^de^ graad: grootouders; 3^de^ graad: ooms, tantes, neven en nichten) van u die reeds appendicitis (ontsteking van de blindedarm) hebben ontwikkeld?

- - Ja, 🞏 1^ste^ graad; 🞏 2^de^ graad; 🞏 3^de^ graad

(duid aan wat van toepassing is door het juiste vakje aan te kruisen)

- - Neen (Ga naar **DAR 16**)

**DAR 15** Ging dit gepaard met eventuele complicaties (vorming van abcessen, perforatie, ed.)?

- - Ja
  - Neen

**DAR 16** Zijn er naaste bloedverwanten (1^ste^ graad: vader, moeder, broers en zussen; 2^de^ graad: grootouders; 3^de^ graad: ooms, tantes, neven en nichten) van u die lijden aan een inflammatoire darmziekte (ziekte van Crohn of Colitis Ulcerosa)?

- - Ja, ziekte van Crohn
  - Ja, Colitis Ulcerosa
  - Neen
  - Weet ik niet

| **SECTIE B3 : Overige vragen ivm gezondheid ed.**  In deze sectie polsen wij naar **specifieke vragen** die verband houden met **ziekte en gezondheid**. Al de informatie die wij van u ontvangen zal vertrouwelijk blijven en dusdanig behandeld worden. |
| --- |

**SPEC 1** Kreeg u als baby borstvoeding?

- - Ja
  - Neen (Ga naar **SPEC 3**)
  - Weet ik niet (Ga naar **SPEC 3**)

**SPEC 2**  Hoelang heeft u als baby borstvoeding gekregen?

- - < 3 maanden
  - 3 – 6 maanden
  - > 6 maanden
  - Weet ik niet

**SPEC 3** Had u in uw jeugd last van astma?

- - Ja
  - Neen
  - Weet ik niet

**SPEC 4** Had u in uw jeugd last van hooikoorts?

- - Ja
  - Neen
  - Weet ik niet

**SPEC 5** Had u in uw jeugd last van eczeem?

- - Ja
  - Neen
  - Weet ik niet

**SPEC 6** Gebruikt u vaak antibiotica?

- - Ja (meer dan 1x per maand)
  - Soms (1x per maand)
  - Zelden
  - Nooit

**SPEC 7**  Gebruikt u vaak probiotica (yoghurt, actieve bifidus, etc.)?

- - Ja, dagelijks
  - 1x per week
  - 1x per maand
  - Zelden
  - Nooit

**SPEC 8**  Heeft u de afgelopen maand, vóór u klachten kreeg van appendicitis, koorts gehad?

- - Ja
  - Neen

**SPEC 9** Heeft u de afgelopen maand, vóór u klachten kreeg van appendicitis, een infectie gehad?

- - Ja; Welke: (bv. bronchitis, griep, blaasontsteking, …)

……………………………………………………………………………………………………………

- - Neen

**SPEC 10** Heeft u de afgelopen maand, vóór u klachten kreeg van appendicitis, antibiotica genomen?

- - Ja;

Welke:…………………………………………………………………………………………………..

Hoeveel dagen:……………………………………………………………………………………..
 Hoeveel keer per dag:……………………………………………………………………………

- - Neen

**SPEC 11** Zijn er naaste bloedverwanten (1^ste^ graad: vader, moeder, broers en zussen;
 2^de^ graad: grootouders) die astma hebben?

- - Ja, 🞏 1^ste^ graad; 🞏 2^de^ graad

(duid aan wat van toepassing is door het juiste vakje aan te kruisen)

- - Neen
  - Weet ik niet

**SPEC 12** Zijn er naaste bloedverwanten (1^ste^ graad: vader, moeder, broers en zussen; 2^de^ graad: grootouders) die last hebben van andere allergieën?

- - Ja;
     Welke:………………………………………………………………………………………………
  - Neen
  - Weet ik niet

**SPEC 13** Heeft u reeds operatief uw keel- (tonsillectomie) en/of neusamandelen (adenotomie) laten verwijderen?

- - Ja, 🞏 tonsillectomie; 🞏 adenotomie
  - Neen
  - Weet ik niet

| **SECTIE C : VOEDING**  In deze sectie vragen wij u om vragen te beantwoorden over **uw eetgewoontes en voedingspatronen**. Al de informatie die wij van u ontvangen zal vertrouwelijk blijven en dusdanig behandeld worden. |
| --- |

**VOE 1** Bent u vegetariër (geen vlees) of veganist (geen vlees en andere dierlijke producten, zoals kaas, melk, eieren, …)?

- - Ja (Ga naar **VOE 3**)
  - Neen

**VOE 2** Hoe vaak eet u vlees? Dit mag alle soorten vlees zijn.

- - Dagelijks
  - Wekelijks
  - Maandelijks
  - Weet ik niet

**VOE 3** Hoe vaak eet u fruit? Dit mag zowel vers fruit, als ingevroren, als in blik zijn.

- - Dagelijks (Aantal stuks:………………………………………..)
  - Wekelijks (Aantal stuks:……………………………………….)
  - Maandelijks (Aantal stuks:……………………………………)
  - Nooit
  - Weet ik niet

**VOE 4** Hoe vaak eet u groenten?

- - Dagelijks (Aantal porties:…………………………………………………)
  - Wekelijks (Aantal porties:………………………………………………..)
  - Maandelijks (Aantal porties:…………………………………………….)
  - Nooit
  - Weet ik niet

**VOE 5** Hoe vaak eet u bonen, erwten, kolen (bloemkool, savooikool, witte kool, …), broccoli en andere vezelbevattende groenten?

- - Dagelijks (Aantal porties:………………………………………………….)
  - Wekelijks (Aantal porties:…………………………………………………)
  - Maandelijks (Aantal porties:…………………………………………….)
  - Nooit
  - Weet ik niet

**VOE 6**  Hoe vaak gebruikt u suikerbevattende dranken? (koud: Coca Cola, Sprite, Fanta, Ice-tea, Nestea, energiedrankjes zoals Red Bull, …) (warm: cappuccino, koffie met suiker, lattes, …)

**Opgelet: Espresso zwart bevat 0 gram suiker en dient niet meegeteld te worden!**

- - Wekelijks (Aantal glazen/koppen:………………………………………….)
  - Maandelijks (Aantal glazen/koppen:………………………………………)
  - Nooit
  - Weet ik niet

**VOE 7** Hoeveel bedraagt uw dagelijkse alcoholcontrole (wijn, bier, … ; geen sterke dranken!)?

- - 0 glazen per dag
  - 1 – 2 glazen per dag
  - 3 – 4 glazen per dag
  - 5 – 6 glazen per dag
  - > 6 glazen per dag
  - Weet ik niet

| **SECTIE D : BEWEGING**  In deze sectie vragen wij u om vragen te beantwoorden over uw **sportieve activiteiten** . Al de informatie die wij van u ontvangen zal vertrouwelijk blijven en dusdanig behandeld worden. |
| --- |

**BEW 1** Hoe vaak per week doet u aan lichamelijke activiteiten ?

- - Niet
  - 1x per week
  - 2 tot 3x per week
  - 5x per week
  - Meer dan 5x per week

**BEW 2** Heeft u veel beweging tijdens uw werk(uren)?

- - Ik heb zittend werk
  - Ik heb zittend werk, maar wandel/fiets wel naar het werk
  - Ik heb werk waarvoor ik vaak actief moet bewegen (trappen lopen,

fietsen, wandelen, …)

- - Ik heb geen werk (werkzoekende)

| **SECTIE E : ROKEN en SECUNDAIR ROKEN**  In deze sectie vragen wij u om vragen te beantwoorden omtrent **uw rokersstatus**. Al de informatie die wij van u ontvangen zal vertrouwelijk blijven en dusdanig behandeld worden. |
| --- |

**ROK 1** Bent u een roker of een roker geweest?

- - Huidige roker
  - Roker in het verleden (Ga naar **ROK 5**)
  - Nooit gerookt (Ga naar **ROK 8**)

**ROK 2** Op welke leeftijd bent u begonnen met roken?

- - 10 – 14 jaar
  - 15 – 24 jaar
  - 25 – 44 jaar
  - 45 – 64 jaar
  - 65+

**ROK 3** Hoe vaak rookt u op dit moment?

- - Dagelijks
  - Occasioneel (tenminste 1 sigaret in 4 weken) (Ga naar **ROK 8**)

**ROK 4**  Hoeveel sigaretten, sigaren, cigarillos, … (e.a. rookwaren) rookt u gemiddeld per dag?

- - 1 – 10
  - 10 – 15 (+/- half pakje)
  - 15 – 20 (+/- 1 pakje)
  - >20

**ROK 5** Hoelang geleden bent u gestopt met roken?

- - Aantal weken (Aantal:……………………..)
  - Aantal maanden (Aantal:……………………)
  - Aantal jaren (Aantal:…………………………)
  - > 10 jaar (Ga naar **ROK 8**)
  - Ik weet het niet

**ROK 6** Tijdens de jaren dat u heeft gerookt, hoe vaak rookte u toen?

- - Dagelijks
  - Occasioneel (tenminste 1 sigaret in 4 weken) (Ga naar **ROK 8**)

**ROK 7** Tijdens de jaren dat u heeft gerookt, hoeveel sigaretten, sigaren, cigarillos, … (e.a. rookwaren) rookte u toen gemiddeld per dag?

- - 1 – 10
  - 10 – 15 (+/- half pakje)
  - 15 – 20 (+/- 1 pakje)
  - >20

**ROK 8** Hoe vaak rookt iemand bij u binnen in huis?

- - Dagelijks
  - Wekelijks
  - Maandelijks
  - Jaarlijks
  - Nooit
  - Weet ik niet
