# Supplementary material for: An observational study on lifestyle and environmental risk factors in patients with acute appendicitis
Source: Heliyon. 2023 Apr 1;9(4):e15131. doi: 10.1016/j.heliyon.2023.e15131 (PMC10147974; doi:10.1016/j.heliyon.2023.e15131)
Supplement: Multimedia component 3 [file mmc3.doc]

| **HAPPIEST cohort Studie Vragenlijst: LIFESTYLE Pk1 (5 t.e.m. 15 jaar)** |
| --- |

Geachte Mijnheer / Mevrouw,

Uw kind doet mee aan de **‘HAPPIEST studie’**, een studie naar de risicofactoren van acute appendicitis en de daarbij mogelijk optredende complicaties. In de informatiebrochure die u en uw kind reeds van de behandelende arts hebben ontvangen, vindt u meer gedetailleerde informatie over de studie.

Wij vragen patiënten die meedoen aan de studie deze vragenlijst in te vullen. Indien de patiënt te jong is om deze vragenlijst alleen in te vullen, vragen wij de ouders om samen met hun kind deze lijst in te vullen. Wij vragen daarbij steeds naar **de eigen mening**. Er zijn geen goede of foute antwoorden. Wij zullen zorgvuldig met deze vertrouwelijke informatie omgaan. Het invullen duurt ongeveer **10-15 minuten**.

De ingevulde **vragenlijst** dient overhandigd te worden **voor het verlaten van het ziekenhuis aan het verpleegkundig personeel op de afdeling**. Als u en uw kind vragen heeft over deze vragenlijst, aarzel dan niet om contact met ons op te nemen (zie contactpersonen, bijlage patiënt informatiebrochure).

Wij willen u en uw kind alvast hartelijk bedanken voor het invullen van deze vragenlijst en voor deelname aan de **HAPPIEST studie**!

Met vriendelijke groet,

Namens het ‘HAPPIEST studie’ team

Prof. Dr. I.C. Gyssens, internist-infectioloog / HAPPIEST Prinicipal investigator (PI)

[i.gyssens@aig.umcn.nl](mailto:i.gyssens@aig.umcn.nl) | [inge.gyssens@jessazh.be](mailto:inge.gyssens@jessazh.be) | +32 11 30 94 85

Jessa Ziekenhuis – Campus Virga Jesse

Stadsomvaart 11

3500 Hasselt

België

| **HAPPIEST cohort Studie Vragenlijst: LIFESTYLE Pk1 (5 t.e.m. 15 jaar)** |
| --- |

| Datum: ………/………/………  Naam: …......................................................  Voornaam: …......................................................  Geboortedatum: ………/………/………  Telefoon/GSM nummer:…......................................................  E-mailadres: …......................................................  Studienummer: …............................... (in te vullen door onderzoeker/studiemedewerker) |
| --- |

| **SECTIE A : Demografische gegevens**  In deze sectie willen wij vragen **uzelf te beschrijven** aan de hand van een paar vragen.  Al de informatie die wij van u ontvangen zal vertrouwelijk blijven en dusdanig behandeld worden. |
| --- |

**DEM 1** Wat is uw geslacht?

- - Man
  - Vrouw

**DEM 2** Wat is uw leeftijd?

- - 5 – 9 jaar
  - 10 – 15 jaar

**DEM 3**  Hoeveel broers/zussen heeft u en wat is hun leeftijd?

1)…………………………………………………………………………………………………………………

2)…………………………………………………………………………………………………………………

3)…………………………………………………………………………………………………………………

4)…………………………………………………………………………………………………………………

5)…………………………………………………………………………………………………………………

6)…………………………………………………………………………………………………………………

7)…………………………………………………………………………………………………………………

**DEM 4** Wat is het hoogste diploma dat u, tot nu toe heeft behaald?

(Opgelet: indien u nog naar school gaat, kleurt u het laatste bolletje)

- - Lager onderwijs
  - Middelbaar onderwijs
  - Niet van toepassing

**DEM 5** Wat is uw etnische achtergrond?

- - Europees
  - Noord-Afrika
  - Sub-Saharan Afrika
  - Aziatisch

**DEM 6** Hoe kan u het gebied omschrijven waar u nu woont?

- - Platteland (landelijk)
  - Stad en/of dorpskern (verstedelijkt)

**DEM 7** Heeft u op een boerderij / hoeve gewoond?

- - Ja
  - Neen

**DEM 8** Komt u vaak in contact met dieren (vee: varkens, koeien, schapen, geiten, paarden, kippen, etc.)?

- - Dagelijks
  - 1 x per maand
  - Zelden
  - Nooit

**DEM 9** Heeft u huisdieren die bij u in huis leven (katten, honden, hamsters, cavia’s, etc.)?

- - Ja
  - Neen

| **SECTIE B1 : Gezondheid**  In deze sectie polsen wij naar **uw algemene gezondheid**. Al de informatie die wij van u ontvangen zal vertrouwelijk blijven en dusdanig behandeld worden. |
| --- |

**GEZ 1** Hoe vindt u uw gezondheid in het algemeen?

- - Uitstekend
  - Zeer goed
  - Goed
  - Redelijk
  - Slecht

**GEZ 2** Hoe vaak had u de afgelopen week klachten over uw lichamelijke gezondheid?

- - Voortdurend
  - Het grootste deel van de tijd
  - Een deel van de tijd
  - Een klein deel van de tijd
  - Geheel niet

**GEZ 3** Hoe vaak had u de afgelopen week klachten over uw mentale gezondheid (zoals stress, angstige en depressieve gevoelens)?

- - Voortdurend
  - Het grootste deel van de tijd
  - Een deel van de tijd
  - Een klein deel van de tijd
  - Geheel niet

**GEZ 4** Hoe vaak heeft uw lichamelijke en/of mentale gezondheid u de afgelopen week belemmerd in het uitvoeren van uw dagelijkse activiteiten, zoals werk, ontspanning, etc.?

- - Voortdurend
  - Het grootste deel van de tijd
  - Een deel van de tijd
  - Een klein deel van de tijd
  - Geheel niet

**GEZ 5**  Hoe vaak heeft u zich de afgelopen week moe of vermoeid gevoeld?

- - Voortdurend
  - Het grootste deel van de tijd
  - Een deel van de tijd
  - Een klein deel van de tijd
  - Geheel niet

| **SECTIE B2 : Algemene gesteldheid van het darmstelsel**  In deze sectie polsen wij naar de **algemene gesteldheid van uw darmstelsel**. Al de informatie die wij van u ontvangen zal vertrouwelijk blijven en dusdanig behandeld worden. |
| --- |

**DAR 1** Hoe vaak heeft u de afgelopen week buikpijn gehad?

- - Voortdurend
  - Het grootste deel van de tijd
  - Een deel van de tijd
  - Een klein deel van de tijd
  - Geheel niet

**DAR 2** Hoe vaak heeft u in de afgelopen week een opgeblazen gevoel (gevoel van teveel lucht in de buik) gehad?

- - Voortdurend
  - Het grootste deel van de tijd
  - Een deel van de tijd
  - Een klein deel van de tijd
  - Geheel niet

**DAR 3** Hoe vaak heeft u in de afgelopen week last gehad van winderigheid?

- - Voortdurend
  - Het grootste deel van de tijd
  - Een deel van de tijd
  - Een klein deel van de tijd
  - Geheel niet

**DAR 4** Hoe vaak heeft u in de afgelopen week last gehad van rommelingen in de buik?

- - Voortdurend
  - Het grootste deel van de tijd
  - Een deel van de tijd
  - Een klein deel van de tijd
  - Geheel niet

**DAR 5** Hoe vaak heeft u er in de afgelopen week last van gehad dat u veelvuldig ontlasting had (vaker naar het toilet gaan dan anders)?

- - Voortdurend
  - Het grootste deel van de tijd
  - Een deel van de tijd
  - Een klein deel van de tijd
  - Geheel niet

**DAR 6** Hoe vaak heeft u in de afgelopen week last gehad van ernstige aandrang tot het krijgen van ontlasting?

- - Voortdurend
  - Het grootste deel van de tijd
  - Een deel van de tijd
  - Een klein deel van de tijd
  - Geheel niet

**DAR 7**  Hoe vaak heeft u in de afgelopen week last gehad van dunnere ontlasting
(diarree)?

- - Voortdurend
  - Het grootste deel van de tijd
  - Een deel van de tijd
  - Een klein deel van de tijd
  - Geheel niet

**DAR 8**  Hoe vaak heeft u in de afgelopen week last gehad van verstopping
(constipatie) ?

- - Voortdurend
  - Het grootste deel van de tijd
  - Een deel van de tijd
  - Een klein deel van de tijd
  - Geheel niet

**DAR 9** Hoe vaak heeft u in de afgelopen week last gehad van misselijkheid?

- - Voortdurend
  - Het grootste deel van de tijd
  - Een deel van de tijd
  - Een klein deel van de tijd
  - Geheel niet

**DAR 10** Wanneer heeft u het eerste duidelijke symptoom van appendicitis (navelpijn, pijn in de rechteronderbuik) ervaren?

- - Vandaag
  - Vannacht
  - Gisteren
  - 2 dagen geleden
  - > 2 dagen geleden

**DAR 11** Wanneer heeft u voor het eerst uw huisarts of een arts geraadpleegd?

- - Vandaag
  - Vannacht
  - Gisteren
  - 2 dagen geleden
  - > 2 dagen geleden

**DAR 12** Zijn er naaste bloedverwanten (1^ste^ graad: vader, moeder, broers en zussen; 2^de^ graad: grootouders; 3^de^ graad: ooms, tantes, neven en nichten) van u die reeds appendicitis (ontsteking van de blindedarm) hebben ontwikkeld?

- - Ja, 🞏 1^ste^ graad; 🞏 2^de^ graad; 🞏 3^de^ graad

(duid aan wat van toepassing is door het juiste vakje aan te kruisen)

- - Neen (Ga naar **DAR 14**)

**DAR 13** Ging dit gepaard met eventuele complicaties (vorming van abcessen, perforatie, ed.)?

- - Ja
  - Neen

**DAR 14** Zijn er naaste bloedverwanten (1^ste^ graad: vader, moeder, broers en zussen; 2^de^ graad: grootouders; 3^de^ graad: ooms, tantes, neven en nichten) van u die lijden aan een inflammatoire darmziekte (ziekte van Crohn of colitis ulcerosa)?

- - Ja, ziekte van Crohn
  - Ja, colitis ulcerosa
  - Neen
  - Weet ik niet

| **SECTIE B3 : Overige vragen ivm gezondheid ed.**  In deze sectie polsen wij naar **specifieke vragen** die verband houden met **ziekte en gezondheid**. Al de informatie die wij van u ontvangen zal vertrouwelijk blijven en dusdanig behandeld worden. |
| --- |

**SPEC 1** Kreeg u als baby borstvoeding?

- - Ja
  - Neen (Ga naar **SPEC 3**)
  - Weet ik niet (Ga naar **SPEC 3**)

**SPEC 2**  Hoelang heeft u als baby borstvoeding gekregen?

- - < 3 maanden
  - 3 – 6 maanden
  - > 6 maanden
  - Weet ik niet

**SPEC 3** Heeft u last van astma?

- - Ja
  - Neen
  - Weet ik niet

**SPEC 4** Heeft u last van hooikoorts?

- - Ja
  - Neen
  - Weet ik niet

**SPEC 5** Heeft u last van eczeem?

- - Ja
  - Neen
  - Weet ik niet

**SPEC 6** Gebruikt u vaak antibiotica?

- - Ja (meer dan 1x per maand)
  - Soms (1x per maand)
  - Zelden
  - Nooit

**SPEC 7**  Gebruikt u vaak probiotica (yoghurt, actieve bifidus, etc.)?

- - Ja, dagelijks
  - 1x per week
  - 1x per maand
  - Zelden
  - Nooit

**SPEC 8**  Heeft u de afgelopen maand, vóór u klachten kreeg van appendicitis, koorts gehad?

- - Ja
  - Neen

**SPEC 9** Heeft u de afgelopen maand, vóór u klachten kreeg van appendicitis, een infectie gehad?

- - Ja; Welke: (bv. bronchitis, griep, blaasontsteking, …)

……………………………………………………………………………………………………………

- - Neen

**SPEC 10** Heeft u de afgelopen maand, vóór u klachten kreeg van appendicitis, antibiotica genomen?

- - Ja;

Welke:…………………………………………………………………………………………………..

Hoeveel dagen:……………………………………………………………………………………..
 Hoeveel keer per dag:……………………………………………………………………………

- - Neen

**SPEC 11** Zijn er naaste bloedverwanten (1^ste^ graad: vader, moeder, broers en zussen;
 2^de^ graad: grootouders) die astma hebben?

- - Ja, 🞏 1^ste^ graad; 🞏 2^de^ graad

(duid aan wat van toepassing is door het juiste vakje aan te kruisen)

- - Neen
  - Weet ik niet

**SPEC 12** Zijn er naaste bloedverwanten (1^ste^ graad: vader, moeder, broers en zussen; 2^de^ graad: grootouders) die last hebben van allergieën (astma, hooikoorts, eczeem)?

- - Ja;
     Welke:………………………………………………………………………………………………
  - Neen
  - Weet ik niet

**SPEC 13** Heeft u reeds operatief uw keel- (tonsillectomie) en/of neusamandelen (adenotomie) laten verwijderen?

- - Ja, 🞏 tonsillectomie; 🞏 adenotomie
  - Neen
  - Weet ik niet

| **SECTIE C : VOEDING**  In deze sectie vragen wij u om vragen te beantwoorden over **uw eetgewoontes en voedingspatronen**. Al de informatie die wij van u ontvangen zal vertrouwelijk blijven en dusdanig behandeld worden. |
| --- |

**VOE 1** Bent u vegetariër (geen vlees) of veganist (geen vlees en andere dierlijke producten, zoals kaas, melk, eieren, …)?

- - Ja (Ga naar **VOE 3**)
  - Neen

**VOE 2** Hoe vaak eet u vlees? Dit mag alle soorten vlees zijn.

- - Dagelijks
  - Wekelijks
  - Maandelijks
  - Weet ik niet

**VOE 3** Hoe vaak eet u fruit? Dit mag zowel vers fruit, als ingevroren, als in blik zijn.

- - Dagelijks (Aantal stuks:………………………………………..)
  - Wekelijks (Aantal stuks:……………………………………….)
  - Maandelijks (Aantal stuks:……………………………………)
  - Nooit
  - Weet ik niet

**VOE 4** Hoe vaak eet u groenten?

- - Dagelijks (Aantal porties:…………………………………………………)
  - Wekelijks (Aantal porties:………………………………………………..)
  - Maandelijks (Aantal porties:…………………………………………….)
  - Nooit
  - Weet ik niet

**VOE 5** Hoe vaak eet u bonen, erwten, kolen (bloemkool, savooikool, witte kool, …), broccoli en andere vezelbevattende groenten?

- - Dagelijks (Aantal porties:………………………………………………….)
  - Wekelijks (Aantal porties:…………………………………………………)
  - Maandelijks (Aantal porties:…………………………………………….)
  - Nooit
  - Weet ik niet

**VOE 6**  Hoe vaak gebruikt u suikerbevattende dranken (koud: Coca Cola, Sprite, Fanta, Ice- tea, Nestea, Fristi,… en/of warm: warme chocomelk, …)?

- - Wekelijks (Aantal glazen/koppen:………………………………………….)
  - Maandelijks (Aantal glazen/koppen:………………………………………)
  - Nooit
  - Weet ik niet

| **SECTIE D : BEWEGING**  In deze sectie vragen wij u om vragen te beantwoorden over uw **sportieve activiteiten** . Al de informatie die wij van u ontvangen zal vertrouwelijk blijven en dusdanig behandeld worden. |
| --- |

**BEW 1** Hoe vaak per week doet u aan lichamelijke activiteiten (turnen, zwemmen, lopen, voetballen, dansen, sporten, …) ?

- - Niet
  - 1x per week
  - 2 tot 3x per week
  - 5x per week
  - Meer dan 5x per week

**BEW 2** Heeft u veel beweging buiten de schooluren (wandelen/fietsen naar school)?

- - Neen
  - Ja
