# Supplementary material for: An observational study on lifestyle and environmental risk factors in patients with acute appendicitis
Source: Heliyon. 2023 Apr 1;9(4):e15131. doi: 10.1016/j.heliyon.2023.e15131 (PMC10147974; doi:10.1016/j.heliyon.2023.e15131)
Supplement: Multimedia component 4 [file mmc4.doc]

500FG

sticker

# Vragenlijst gezondheid

voor deelnemers van 18 tot 75 jaar

Deze vragenlijst omvat vragen die te maken hebben met uw gezondheid en is onderdeel van het 500 Functional Genomics project. Het invullen van de vragenlijst zal ongeveer 20-30 minuten duren.

**
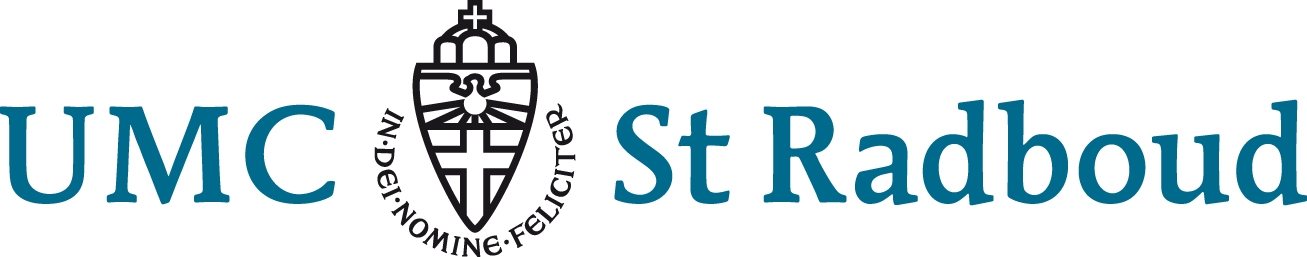
**


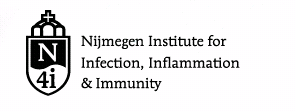

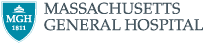

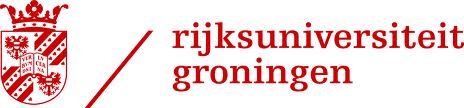


# Vragenlijst gecontroleerd door:

Geachte Meneer / Mevrouw,

U doet mee aan het **‘500FG project’**, een studie naar of het wel of niet aanwezig zijn van een bepaalde groep huid-of darmflora invloed kan hebben op de ontwikkeling van verschillende ziektes. Ook kan de aanwezigheid van een bepaald soort bloedcellen een belangrijke rol spelen. In de informatiebrochure die u reeds van de onderzoeker of een studiemedewerker heeft ontvangen, vindt u meer gedetailleerde informatie over de studie.

Wij vragen deelnemers die meedoen aan de studie deze vragenlijst in te vullen. Wij vragen u daarbij steeds om **uw eigen mening**. Er zijn geen goede of foute antwoorden. Wij zullen zorgvuldig met deze vertrouwelijke informatie omgaan. Het invullen duurt ongeveer **20-30 minuten**.

De vragenlijst dient u na het invullen terug te overhandigen aan de onderzoeker of aan een studiemedewerker. Als u vragen heeft over deze vragenlijst, aarzel dan niet om contact met ons op te nemen (zie contactpersonen, informatiebrochure).

Wij willen u alvast hartelijk danken voor het invullen van deze vragenlijst en uw deelname aan het **500FG project**!

Met vriendelijke groet,

Het ‘500FG-team’

**Wilt u voordat u begint met het beantwoorden van de vragen de volgende punten doorlezen?**

**Lees eerst de vraag en antwoordmogelijkheden.**

Vul daarna het rondje van het juiste antwoord volledig in. Goed **•** Fout ○ ○

**Er is per vraag maar 1 antwoord mogelijk tenzij anders vermeld.**

Vul de hele vragenlijst in en sla alstublieft geen vragen over! Wanneer u het antwoord niet precies weet mag u een schatting geven. Op de laatste pagina van de vragenlijst kunt u eventuele twijfel over juistheid van antwoorden aangeven.

**U hebt een fout gemaakt?**

Als u een fout maakt is dat niet erg. Zet een kruis door het foute antwoord en vul het rondje van het goede antwoord. Hieronder staat een voorbeeld.

Hebt u een bril of contactlenzen nodig?

**•** Ja, alleen voor dichtbij (lezen e.d.)

○ Ja, alleen om veraf te kijken (autorijden, televisiekijken e.d.)

**•** Ja, zowel voor dichtbij als voor veraf

○ Nee

(Het goede antwoord is dus Ja, zowel voor dichtbij als veraf)

**SECTIE A : Demografische gegevens**

In deze sectie willen wij vragen **uzelf te beschrijven** aan de hand van een paar vragen.

Al de informatie die wij van u ontvangen zal vertrouwelijk blijven en dusdanig behandeld worden.

**Wilt u allereerst de datum van vandaag invullen**: _ _ - _ _ - _ _ _ _ (dag-maand-jaar)

**DEM 1** Wat is uw geslacht?

- - Man
  - Vrouw

**DEM 2** Wat is uw leeftijd? _ _ _ _ _ _ jaar

**DEM 3** Wat is uw lengte? _ _ _ _ _ _ meter

Wat is uw gewicht? _ _ _ _ _ _ kg

**DEM 4** Wat is uw huidige burgerlijke staat? (Gelieve één te kiezen die het beste uw huidige situatie beschrijft)

- - Alleen wonend
  - Samenwonend met partner of familieli(e)d(en)
  - Wonend in gemeenschap (studentenhuis, rusthuis, instelling, …)

**DEM 5** Wat is het hoogste diploma dat u, tot nu toe heeft behaald?

(Opgelet: indien u nog naar school gaat, kleurt u het laatste bolletje)

- - Lager onderwijs
  - Middelbaar beroepsonderwijs (MBO)
  - Hogeschool opleiding (HBO)
  - Universitaire opleiding
  - Ik ben nog in opleiding (Specifiek:………………………………………………………)

**DEM 6** Wat voor werk doet u op dit moment / wat is uw beroep?

……………………………………………………………………………

**DEM 7** Wat is uw etnische achtergrond?

- - Europees
  - Noord-Afrika
  - Sub-Saharan Afrika
  - Aziatisch

**DEM 8** Hoe kan u het gebied omschrijven waar u nu woont?

- - Platteland (landelijk)
  - Stad en/of dorpskern (verstedelijkt)

**DEM 9**  Hoe kan u het gebied omschrijven waar u in uw jeugd heeft gewoond?

- - Platteland (landelijk)
  - Stad en/of dorpskern (verstedelijkt)

**DEM 10** Kwam u in uw jeugd vaak in contact met boerderijdieren (varkens, koeien, schapen, geiten, paarden, kippen, etc.)

- - Dagelijks
  - 1 x per maand
  - Zelden
  - Nooit

**DEM 11** Heeft u op dit moment huisdieren die bij u in huis leven (katten, honden, hamsters, cavia’s, etc.)?

- - Ja
  - Nee

**DEM 12** Bent u de afgelopen 10 jaar buiten Europa geweest?

- - Ja, welke landen en wanneer: …………………………………………………………… ……………………………………………………………………………………………………………………………………
  - Nee

**DEM 13** Wat is uw seksuele geaardheid?

- - Heterosexueel
  - Bisexueel
  - Homosexueel

**DEM 14** Hoeveel seksuele contacten had u het afgelopen jaar?

- - Geen partner
  - Één vaste partner
  - Wisselende partners

| **SECTIE B1 : Gezondheid**  In deze sectie polsen wij naar **uw algemene gezondheid**. Al de informatie die wij van u ontvangen zal vertrouwelijk blijven en dusdanig behandeld worden. |
| --- |

**GEZ 1** Hoe vindt u uw gezondheid in het algemeen?

- - Uitstekend
  - Zeer goed
  - Goed
  - Redelijk
  - Slecht

**GEZ 2** Hoe vaak had u de afgelopen 3 weken klachten over uw lichamelijke gezondheid?

- - Voortdurend
  - Het grootste deel van de tijd
  - Een deel van de tijd
  - Een klein deel van de tijd
  - Geheel niet

**GEZ 3** Hoe vaak had u de afgelopen 3 weken klachten over uw mentale gezondheid (zoals stress, angstige en depressieve gevoelens)?

- - Voortdurend
  - Het grootste deel van de tijd
  - Een deel van de tijd
  - Een klein deel van de tijd
  - Geheel niet

**GEZ 4** Gebruikt u medicijnen?

- - Nee
  - Ja, welke en dosering:…………………………………………………………………………

…………………………………………………………………………………………………………………………

**GEZ 5** Gebruikt u de pil als anticonceptiemiddel?

- - Ja
  - Nee
  - Nee, ik ben een man

**GEZ 6** Heeft u hartproblemen?

- - Ja
  - Nee

**GEZ 7** Heeft u problemen met uw bloedvaten (b.v. beroerte, etalagebenen)?

- - Ja
  - Nee

**GEZ 8** Is bij u de afgelopen 10 jaar een verhoogde bloeddruk gemeten?

- - Ja
  - Nee

**GEZ 9** Is er bij u ooit een verhoogd cholesterol gevonden?

- - Ja
  - Nee

**GEZ 10** Heeft u last van drukkende pijn op de borst bij inspanning?

- - Ja
  - Nee

**GEZ 11** Heeft u last van pijn in uw benen bij het lopen?

- - Ja
  - Nee

**GEZ 12** Heeft u suikerziekte?

- - Ja
  - Nee

**GEZ 13** Heeft u een nierziekte?

- - Ja, welke:……………………………………………………………….
  - Nee

**GEZ 14** Heeft of had uw vader, moeder, broer of zus:

- - Ja, specificeer:

Welk familielid Hoe oud waren ze bij diagnose

Hartaanval ……………………… ………………………………………………..

Angina pectoris ……………………… ………………………………………………..

Beroerte ……………………… ………………………………………………..

Nierziekte ……………………… ………………………………………………..

Suikerziekte ……………………… ………………………………………………..

- - Nee

| **SECTIE B2 : Algemene gesteldheid van het darmstelsel**  In deze sectie polsen wij naar de **algemene gesteldheid van uw darmstelsel**. Al de informatie die wij van u ontvangen zal vertrouwelijk blijven en dusdanig behandeld worden. |
| --- |

**DAR 1** Hoe vaak heeft u de laatste 3 maanden klachten of buikpijn gehad?

- - Nooit **→ ga verder met SPEC 1 (sectie B3)**
  - Minder dan één dag per maand
  - Eén dag per maand
  - Twee tot drie dagen per maand
  - Eén dag per week
  - Vaker dan één dag per week
  - Elke dag

**DAR 2** Voor vrouwen: had u deze klachten of pijn in uw buik alleen tijdens de menstruatie?

- Nee
- Ja
- Niet van toepassing, omdat ik niet meer menstrueer (menopauze) of ik ben een man

**DAR 3**  Hoe vaak heeft u in de afgelopen week 3 maanden gehad van misselijkheid?

- - Voortdurend
  - Het grootste deel van de tijd
  - Een deel van de tijd
  - Een klein deel van de tijd
  - Geheel niet

**DAR 4** Was in de afgelopen 3 maanden het overgrote deel van uw ontlasting zeer zacht,dun of waterig?

- Ja
  - Nee

**DAR 5** Heeft u reeds appendicitis (ontsteking van de blinde darm) gehad of bent u ooit geopereerd aan uw blinde darm?

- - Ja
  - Nee
  - Weet ik niet

**DAR 6** Zijn er naaste bloedverwanten (1^ste^ graad: vader, moeder, broers en zussen; 2^de^ graad: grootouders; 3^de^ graad: ooms, tantes, neven en nichten) van u die lijden aan een inflammatoire darmziekte (ziekte van Crohn, Colitis Ulcerosa, appendicitis)?

- - Ja, ziekte van Crohn
  - Ja, Colitis Ulcerosa
  - Ja, appendicitis
  - Nee
  - Weet ik niet

| **SECTIE B3 : Overige vragen ivm gezondheid**  In deze sectie polsen wij naar **specifieke vragen** die verband houden met **ziekte en gezondheid**. Al de informatie die wij van u ontvangen zal vertrouwelijk blijven en dusdanig behandeld worden. |
| --- |

**SPEC 1** Kreeg u als baby borstvoeding?

- - Ja
  - Nee **→ ga verder met vraag SPEC 3**
  - Weet ik niet **→ ga verder met vraag SPEC 3**

**SPEC 2**  Hoelang heeft u als baby borstvoeding gekregen (mocht u dit niet weten, maar is het te achterhalen, informeer dit dan bij de onderzoekers)

- - < 3 maanden
  - 3 – 6 maanden
  - > 6 maanden
  - Weet ik niet

**SPEC 3** Had u in uw jeugd last van astma?

- - Ja
  - Nee
  - Weet ik niet

**SPEC 4** Had u in uw jeugd last van hooikoorts?

- - Ja, tot welke leeftijd:………………………………………..
  - Nee
  - Weet ik niet

**SPEC 5** Had u in uw jeugd last van eczeem?

- - Ja
  - Nee
  - Weet ik niet

**SPEC 6** Gebruikt u vaak antibiotica?

- - Ja (meer dan 1x per maand)
  - Soms (1x per maand)
  - Zelden
  - Nooit

**SPEC 7**  Gebruikt u vaak probiotica (activia, yakult, actieve bifidus, etc.)?

- - Ja, dagelijks
  - 1x per week
  - 1x per maand
  - Zelden
  - Nooit

**SPEC 8**  Heeft u een allergie tegen:

- - Geen allergie
  - Noten
  - Zaden
  - Melk of melkproducten
  - Vlees
  - Soja
  - Vis/schelpdieren
  - Eieren
  - Vezels (gluten)
  - Groenten / fruit
  - Anders, namelijk:……………………………………………………………………….

**SPEC 9** Heeft u reeds operatief uw keel- (tonsillectomie) en/of neusamandelen (adenotomie) laten verwijderen?

- - Ja, 🞏 tonsillectomie; 🞏 adenotomie
  - Nee
  - Weet ik niet

**SPEC 10** Heeft u een tekenbeet gehad?

- - Ja
  - Nee **→ ga verder met vraag SPEC15**
  - Weet ik niet **→ ga verder met vraag SPEC15**

**SPEC 11** Hoeveel tekenbeten heeft u gehad?

- - 1
  - 2-5
  - 6-12
  - 12 of meer

**SPEC 12** Heeft u een kring op de huid gehad (Erythema migrans)?

- - Ja, gezien door: 🞏 mijzelf/partner

🞏 huisarts

🞏 specialist: ………………………………………….

- - Nee

**SPEC 13** Bent u ooit behandeld voor ziekte van Lyme?

- - Nee
  - Ja, in welk jaar :…………………………………………..

door wie : huisarts / specialist / beide

antibiotica gehad: ja/nee

welke verschijnselen had u toen? huidafwijking/gewrichtspijnen/hoofdpijn/of anders nl:………………………………………………………………………………………………………..

**SPEC 14** Heeft u momenteel klachten door deze ziekte?

- - Ja,namelijk:…………………………………………………………………………………………………
  - Nee

**SPEC 15** Heeft u momenteel:

Spataderen □ Ja □ nee

Rood of blauw verkleurde onderbenen of voeten □ Ja □ nee

Verschil in dikte of kleur tussen de beide benen of voeten □ Ja □ nee

Verschil in dikte of kleur tussen beide oren □ Ja □ nee

Pijnlijke of dikke gewrichten □ Ja □ nee

Verschijnselen van een hernia

(rugpijn die uitstraalt naar één been) □ Ja □ nee

Doof gevoel in de handen of voeten □ Ja □ nee

Hartritmestoornissen □ Ja □ nee

Geheugenproblemen □ Ja □ nee

Vaak hoofdpijn □ Ja □ nee

Vaak spierpijn □ Ja □ nee

Concentratiestoornissen □ Ja □ nee

**SPEC16** Heeft u wel eens last gehad van schimmelinfecties?

- - Ja, namelijk:…………………………………………………………………………………..
  - Nee
  - Weet ik niet

| **SECTIE C : VOEDING**  In deze sectie vragen wij u om vragen te beantwoorden over **uw eetgewoontes en voedingspatronen**. Al de informatie die wij van u ontvangen zal vertrouwelijk blijven en dusdanig behandeld worden. |
| --- |

**VOE 1** Bent u vegetariër (geen vlees) of veganist (geen vlees en andere dierlijke producten, zoals kaas, melk, eieren, …)?

- - Ja **→ ga verder met vraag**  **VOE 3**
  - Nee

**VOE 2** Hoe vaak eet u vlees? Dit mag alle soorten vlees zijn.

- - Dagelijks
  - Wekelijks
  - Maandelijks
  - Weet ik niet

Wat voor vlees is dit voornamelijk?

- - Rund
  - Varken
  - Kip of ander gevogelte
  - Wild

Hoe vaak eet u vis? Dit mag alle soorten vis zijn.

- - Dagelijks
  - Wekelijks
  - Maandelijks
  - Weet ik niet
  - Ik eet geen vis **→ ga verder met vraag**  **VOE 3**

Wat voor vis is dit voornamelijk?

- - Rauwe vis (bv. Haring)
  - Witte vis (bv. Kabeljauw)
  - Andere vis (bv. Zalm of tonijn)
  - Schelpdieren (bv. Mosselen)

**VOE 3** Hoe vaak eet u fruit? Dit mag zowel vers fruit, als ingevroren, als in blik zijn.

- - Dagelijks (Aantal stuks:………………………………………..)
  - Wekelijks (Aantal stuks:……………………………………….)
  - Maandelijks (Aantal stuks:……………………………………)
  - Nooit
  - Weet ik niet

**VOE 4** Hoe vaak eet u groenten?

- - Dagelijks (Aantal porties:…………………………………………………)
  - Wekelijks (Aantal porties:………………………………………………..)
  - Maandelijks (Aantal porties:…………………………………………….)
  - Nooit
  - Weet ik niet

**VOE 5** Hoe vaak eet u bonen, erwten, kolen (bloemkool, savooikool, witte kool, …), broccoli en andere vezelbevattende groenten?

- - Dagelijks (Aantal porties:………………………………………………….)
  - Wekelijks (Aantal porties:…………………………………………………)
  - Maandelijks (Aantal porties:…………………………………………….)
  - Nooit
  - Weet ik niet

**VOE 6**  Hoe vaak gebruikt u suikerbevattende dranken? (koud: Coca Cola, Sprite, Fanta, Ice- tea, Nestea, energiedrankjes zoals Red Bull, …) (warm: cappuccino, koffie met suiker, lattes, …)

- - Wekelijks (Aantal glazen/koppen:………………………………………….)
  - Maandelijks (Aantal glazen/koppen:………………………………………)
  - Nooit
  - Weet ik niet

**VOE 7** Hoeveel alcohol gebruikt u **doordeweeks** per dag (wijn, bier, … ; geen sterke dranken)?

- - 0 glazen per dag
  - 1 – 2 glazen per dag
  - 3 – 4 glazen per dag
  - 5 – 6 glazen per dag
  - > 6 glazen per dag
  - Weet ik niet

**VOE 8** Hoeveel alcohol gebruikt u in het **weekend** per dag (wijn, bier, … ; geen sterke dranken)?

- - 0 glazen per dag
  - 1 – 2 glazen per dag
  - 3 – 4 glazen per dag
  - 5 – 6 glazen per dag
  - > 6 glazen per dag
  - Weet ik niet

**VOE 9** Hoeveel chocolade eet u per maand?

- - Geen
  - 0-1 reep á 200 gram
  - 1-5 repen á 200 gram
  - 5-10 repen á 200 gram
  - Meer

**VOE 9** Is dit voornamelijk:

- - Puur
  - Melk
  - Wit

**VOE 10** Hoeveel melk drinkt u per week?

- - Geen
  - 1 - 2 bekers per dag (á 200 mL)
  - 2 – 4 bekers per dag (á 200 mL)
  - Meer

**VOE 11** Is deze melk:

- - Gepasteuriseerd
  - Lang houdbaar

| **SECTIE D : BEWEGING**  In deze sectie vragen wij u om vragen te beantwoorden over uw **sportieve activiteiten** . Al de informatie die wij van u ontvangen zal vertrouwelijk blijven en dusdanig behandeld worden. |
| --- |

**BEW 1** Hoe vaak per week doet u aan lichamelijke activiteiten/sporten ?

- - Niet
  - 1x per week
  - 2 tot 3x per week
  - 5x per week
  - Meer dan 5x per week

**BEW 2** Heeft u veel beweging tijdens uw werk(uren)?

- - Ik heb zittend werk
  - Ik heb zittend werk, maar wandel/fiets wel naar het werk
  - Ik heb werk waarvoor ik vaak actief moet bewegen (trappen lopen,

fietsen, wandelen, …)

- - Ik heb zwaar fysiek werk (Tillen, etc)
  - Ik heb geen werk (werkzoekende)

| **SECTIE E : ROKEN en SECUNDAIR ROKEN**  In deze sectie vragen wij u om vragen te beantwoorden omtrent **uw rokersstatus**. Al de informatie die wij van u ontvangen zal vertrouwelijk blijven en dusdanig behandeld worden. |
| --- |

**ROK 1** Bent u een roker of een roker geweest?

- - Huidige roker **→ ga verder met vraag**  **ROK 2**
  - Roker in het verleden **→ ga verder met vraag**  **ROK 4**
  - Nooit gerookt **→ u bent klaar met invullen van de vragenlijst**
  - Iemand in mijn huis rookt

**ROK 2** Op welke leeftijd bent u begonnen met roken? ____________

**ROK 3**  Hoeveel sigaretten, sigaren, cigarillos, … (e.a. rookwaren) rookt u gemiddeld per dag?

………………………………………………………………………………………………..

**ROK 4** Hoelang geleden bent u gestopt met roken?

**……………………………………………………………………………………….**
