# Supplementary material for: An observational study on lifestyle and environmental risk factors in patients with acute appendicitis
Source: Heliyon. 2023 Apr 1;9(4):e15131. doi: 10.1016/j.heliyon.2023.e15131 (PMC10147974; doi:10.1016/j.heliyon.2023.e15131)
Supplement: Multimedia component 5 [file mmc5.doc]

500FG

sticker

# Vragenlijst gezondheid

voor deelnemers van 18 tot 75 jaar

Deze vragenlijst omvat vragen die te maken hebben met uw gezondheid en is onderdeel van het 500 Functional Genomics project. Het invullen van de vragenlijst zal ongeveer 20-30 minuten duren.

**
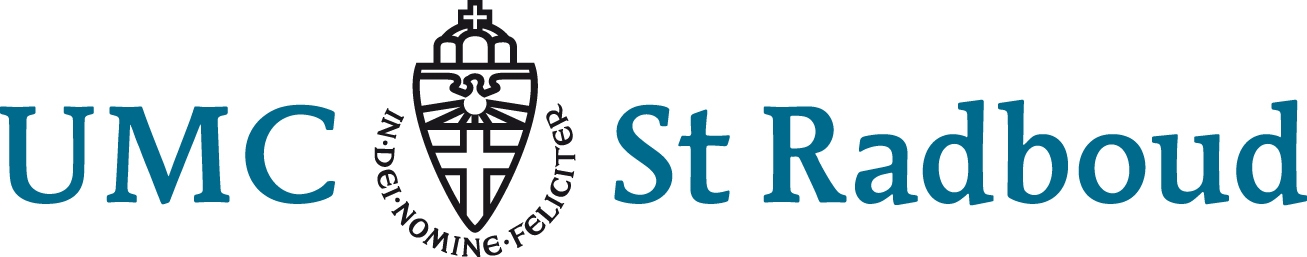
**


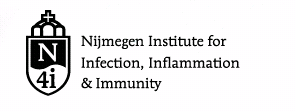

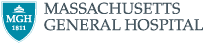

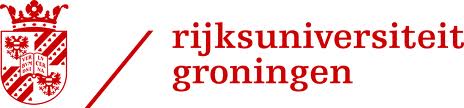


# Vragenlijst gecontroleerd door:

Geachte Meneer / Mevrouw,

U doet mee aan het **‘500FG project’**, een studie naar of het wel of niet aanwezig zijn van een bepaalde groep huid-of darmflora invloed kan hebben op de ontwikkeling van verschillende ziektes. Ook kan de aanwezigheid van een bepaald soort bloedcellen een belangrijke rol spelen. In de informatiebrochure die u reeds van de onderzoeker of een studiemedewerker heeft ontvangen, vindt u meer gedetailleerde informatie over de studie.

Wij vragen deelnemers die meedoen aan de studie deze vragenlijst in te vullen. Wij vragen u daarbij steeds om **uw eigen mening**. Er zijn geen goede of foute antwoorden. Wij zullen zorgvuldig met deze vertrouwelijke informatie omgaan. Het invullen duurt ongeveer **20-30 minuten**.

De vragenlijst dient u na het invullen terug te overhandigen aan de onderzoeker of aan een studiemedewerker. Als u vragen heeft over deze vragenlijst, aarzel dan niet om contact met ons op te nemen (zie contactpersonen, informatiebrochure).

Wij willen u alvast hartelijk danken voor het invullen van deze vragenlijst en uw deelname aan het **500FG project**!

Met vriendelijke groet,

Het ‘500FG-team’

**Wilt u voordat u begint met het beantwoorden van de vragen de volgende punten doorlezen?**

**Lees eerst de vraag en antwoordmogelijkheden.**

Vul daarna het rondje van het juiste antwoord volledig in. Goed **•** Fout ○ ○

**Er is per vraag maar 1 antwoord mogelijk tenzij anders vermeld.**

Vul de hele vragenlijst in en sla alstublieft geen vragen over! Wanneer u het antwoord niet precies weet mag u een schatting geven. Op de laatste pagina van de vragenlijst kunt u eventuele twijfel over juistheid van antwoorden aangeven.

**U hebt een fout gemaakt?**

Als u een fout maakt is dat niet erg. Zet een kruis door het foute antwoord en vul het rondje van het goede antwoord. Hieronder staat een voorbeeld.

Hebt u een bril of contactlenzen nodig?

**•** Ja, alleen voor dichtbij (lezen e.d.)

○ Ja, alleen om veraf te kijken (autorijden, televisiekijken e.d.)

**•** Ja, zowel voor dichtbij als voor veraf

○ Nee

(Het goede antwoord is dus Ja, zowel voor dichtbij als veraf)

**Wilt u allereerst de datum van vandaag invullen**: _ _ - _ _ - _ _ _ _ (dag-maand-jaar)

Zijn er naaste bloedverwanten (1^ste^ graad: vader, moeder, broers en zussen; 2^de^ graad: grootouders; 3^de^ graad: ooms, tantes, neven en nichten) van u die reeds appendicitis (ontsteking van de blindedarm) hebben ontwikkeld?

- - Ja, 🞏 1^ste^ graad; 🞏 2^de^ graad; 🞏 3^de^ graad

(duid aan wat van toepassing is door het juiste vakje aan te kruisen)

- - Neen (Ga naar **DAR 16**)

Ging dit gepaard met eventuele complicaties (vorming van abcessen, perforatie, ed.)?

- - Ja
  - Neen

Zijn er naaste bloedverwanten (1^ste^ graad: vader, moeder, broers en zussen; 2^de^ graad: grootouders; 3^de^ graad: ooms, tantes, neven en nichten) van u die lijden aan een inflammatoire darmziekte (ziekte van Crohn of Colitis Ulcerosa)?

- - Ja, ziekte van Crohn
  - Ja, Colitis Ulcerosa
  - Neen
  - Weet ik niet

Had u in uw jeugd last van eczeem?

- - Ja
  - Neen
  - Weet ik niet
